# Accuracy of heart failure ascertainment using routinely collected healthcare data: a systematic review and meta-analysis

**DOI:** 10.1186/s13643-024-02477-5

**Published:** 2024-03-01

**Authors:** Michelle. A. Goonasekera, Alison Offer, Waseem Karsan, Muram El-Nayir, Amy E. Mallorie, Sarah Parish, Richard J. Haynes, Marion M. Mafham

**Affiliations:** 1 Clinical Trial Service Unit and Epidemiological Studies Unit, Oxford Population Health, University of Oxford, Oxford, UK; 2 Nuffield Department of Population Health, MRC Population Health Research Unit, University of Oxford, Oxford, UK; 3 Clinical Trial Service Unit and Epidemiological Studies Unit, Oxford Population Health, Richard Doll Building, Old Road Campus, Roosevelt Drive, Oxford, OX3 7LF UK

**Keywords:** Randomized trials,, Methods comparison,, Outcome ascertainment,, Streamlined clinical trials,, Systematic review,, Meta-analysis

## Abstract

**Background:**

Ascertainment of heart failure (HF) hospitalizations in cardiovascular trials is costly and complex, involving processes that could be streamlined by using routinely collected healthcare data (RCD). The utility of coded RCD for HF outcome ascertainment in randomized trials requires assessment. We systematically reviewed studies assessing RCD-based HF outcome ascertainment against “gold standard” (GS) methods to study the feasibility of using such methods in clinical trials.

**Methods:**

Studies assessing International Classification of Disease (ICD) coded RCD-based HF outcome ascertainment against GS methods and reporting at least one agreement statistic were identified by searching MEDLINE and Embase from inception to May 2021. Data on study characteristics, details of RCD and GS data sources and definitions, and test statistics were reviewed. Summary sensitivities and specificities for studies ascertaining acute and prevalent HF were estimated using a bivariate random effects meta-analysis. Heterogeneity was evaluated using *I*^2^ statistics and hierarchical summary receiver operating characteristic (HSROC) curves.

**Results:**

A total of 58 studies of 48,643 GS-adjudicated HF events were included in this review. Strategies used to improve case identification included the use of broader coding definitions, combining multiple data sources, and using machine learning algorithms to search free text data, but these methods were not always successful and at times reduced specificity in individual studies. Meta-analysis of 17 acute HF studies showed that RCD algorithms have high specificity (96.2%, 95% confidence interval [CI] 91.5–98.3), but lacked sensitivity (63.5%, 95% CI 51.3–74.1) with similar results for 21 prevalent HF studies. There was considerable heterogeneity between studies.

**Conclusions:**

RCD can correctly identify HF outcomes but may miss approximately one-third of events. Methods used to improve case identification should also focus on minimizing false positives.

**Supplementary Information:**

The online version contains supplementary material available at 10.1186/s13643-024-02477-5.

## Introduction

Heart failure (HF) is an important cause of morbidity and mortality in the general population affecting 1–3% of adults, with over 64 million people estimated to be affected worldwide [1–3]. It is a significant burden on healthcare systems, accounting for about 2% of all healthcare expenditure in countries across Europe and the USA [1, 2]. Therefore, HF is an important target for treatment, requiring large randomized, controlled trials to assess potential interventions. Such large trials can be complex and costly [4, 5]. Ascertainment of HF admissions in a clinical trial often requires clinic visits (with or without manual medical records review) to identify potential events, gathering clinical documents for reported events, and independent clinical adjudication to confirm or refute events. This process could be streamlined to reduce the complexity and overall cost of trials [6–8]. Routinely collected healthcare data (RCD) may help to achieve this goal by supporting the ascertainment of HF outcomes during within-trial periods, and post-trial assessments of the impact on longer-term HF risk [9].

RCD is defined as “healthcare data collected for purposes other than research or without specific a priori research questions developed before collection” [10]. When patients are diagnosed with HF during a healthcare encounter, this diagnosis, along with other data relating to the encounter, are recorded in RCD, usually in the form of coded diagnoses. The most common RCD source is hospital administrative claims data (ACD), an umbrella term for data generated as part of the financial administration of hospitals [11, 12]. Other RCD sources include patient or disease registries and epidemiological surveys (detailed definitions of RCD sources used are provided in Additional file 1: Supplemental Methods). RCD can be used to ascertain events by searching the data for specific codes or coding algorithms.

Ascertaining hospitalizations for HF from such sources can be problematic as HF is a chronic disease with episodes of decompensation requiring admission, and commonly used coding systems do not distinguish between acute events and prevalent chronic disease.

A meta-analysis published in 2014 of 11 studies reporting sensitivity and specificity of coded administrative data for ascertaining HF, showed that pooled sensitivity was 75% (95% confidence interval [CI] 74.7–75.9) and pooled specificity was 97% (95% CI 96.8–96.9) [13]. These findings mirrored two previous reviews [14, 15]. However, there was a limited number of studies in this review, and some studies had very small numbers of HF events. It is also possible that coding practices have improved over the last decade. A systematic review from 2020, focused entirely on Europe and including 20 studies using electronic health records and primary care data, reported sensitivities ≤ 66% and specificities ≥ 95% in most of the studies [16]. However, it excluded other data sources such as claims databases and registries and was geographically restricted. We have systematically reviewed all studies that assessed the utility of RCD for HF outcome ascertainment to summarise the currently available evidence supporting their use in cardiovascular outcomes trials.

## Methods

This review follows the PRISMA (Preferred Reporting for Systematic Reviews and Meta-Analyses) guidelines for conducting and reporting a systematic review [17].

### Search strategy

A search was conducted of all available peer-reviewed literature on MEDLINE and Embase, from their inception (1946 and 1974 respectively), until May 2021 using the Ovid search engine. The initial search strategy was broad and aimed to include any studies where RCD was used to ascertain HF. No limits were set for the initial search. Multiple search terms, including different phrasings or synonyms of the same term were used (see Systematic Review Protocol in the Supplementary Appendix for search strategy and inclusion criteria). After removing duplicates, the titles and abstracts of potentially eligible articles were reviewed and those meeting the inclusion criteria underwent full-text review. The references of the full-text papers were hand-searched for additional relevant articles.

### Inclusion and exclusion criteria

To be included in the review, a study was required to assess the utility of coded RCD for ascertainment of HF against gold standard (GS) ascertainment criteria. We selected full-length, peer-reviewed articles published in English that used RCD to ascertain HF events and reported at least one agreement statistic, or sufficient data to allow its calculation, for International Classification of Disease (ICD) code-based definitions of HF. All studies included must have defined a GS against which to assess the RCD-based ascertainment method and include at least 50 HF events identified using the GS method relevant to that study. The GS method is defined as the reference standard against which each study assessed their RCD-based outcome ascertainment method. Examples include medical records review using pre-specified criteria. Articles were excluded if they used free-text electronic medical records (i.e., narrative clinical notes) as the sole RCD source as these would be considered medical records and are often used as the GS for event adjudication (see Systematic Review Protocol in Supplementary Appendix for detailed exclusion criteria).

### Data extraction

The full-text articles were reviewed by the first author (MAG) who abstracted the data into a data collection form. The author extracted study characteristics, details of the data sources (RCD and GS), type of hospital encounter (e.g., inpatient, outpatient, or emergency department attendances), and data definitions used, along with agreement statistics for the ICD code or coding algorithm used to ascertain HF. The agreement statistics extracted included sensitivity, specificity, positive predictive value (PPV), negative predictive value (NPV), and kappa scores. Where agreement statistics were unavailable, raw data was extracted for calculation where possible. Most routine databases list the main reason for hospitalization (most responsible diagnosis) in a primary position and secondary complications or pre-existing comorbidities in secondary positions. As the distinction between these categories is likely to be important in ascertaining incident episodes of heart failure (e.g., hospitalization due to HF decompensation) as potential trial outcomes, the coding positions and agreement statistics according to coding position were also abstracted where available. If a study used more than one RCD definition or algorithm, the algorithm with the best agreement statistics was used for the main analysis.

Studies were categorized according to which types of RCD-based and GS HF events were included. Studies that only included hospitalizations for decompensated HF, irrespective of a prior HF diagnosis, were categorized as acute HF studies. These studies were the main focus of the analysis as such methods could be used to collect follow-up information in a clinical trial. Studies that included all individuals with HF recorded over the study period (new and pre-existing HF) were categorized as prevalent HF studies. Such methods could be used to identify potential participants for inclusion in clinical trials. Studies that defined HF as a comorbid disease in individuals admitted with another main diagnosis such as myocardial infarction were also included in the prevalent HF category.

If a study assessed both acute and prevalent HF, or different ICD versions, or more than one coding position separately, the agreement statistics were extracted for all relevant event types or RCD algorithms for subgroup analysis. The first author conducted a second review of the abstracted data comparing them against the original abstract to correct any discrepancies in the data collection form. Any uncertainties were resolved through discussion with two senior clinicians (MMM and RJH).

### Study quality assessment

A quality assessment of the included studies was undertaken using the revised tool for Quality Assessment of Diagnostic Accuracy Studies (QUADAS-2) [18]. Three authors (WK, ME, and AEM) independently reviewed the studies and extracted data using the QUADAS-2 template, and the first author reviewed and collated the final quality assessment. Studies were classified as having a low, high, or unclear risk of bias for 4 domains (patient selection, index test, reference standard, and flow and timing) and the first 3 domains were also assessed for applicability to the review question (see Supplemental Methods in Additional file 1 for details). Studies were considered to have a “low risk” of bias or “low concern” regarding applicability if all domains were low risk. If one or more domains had unclear or high risk the study was considered to be “at risk” of bias or have “some concerns” regarding applicability. A sensitivity analysis excluding studies at risk of bias was undertaken.

### Statistical analysis

Studies were grouped according to whether they assessed acute or prevalent HF. Other potential sources of heterogeneity included coding system, position and definitions used, RCD and GS data source, study size, publication date, and country or region (e.g., Europe). All agreement statistics (sensitivity, specificity, PPV or NPV) and 95% CI (exact binomial CI) were calculated using available data (see Additional file 1: Figure S1 for an example 2 × 2 table) [19]. Summary sensitivity, specificity, and a summary receiver operating characteristic (SROC) plot with a summary curve (using the hierarchical SROC model) were obtained using the Stata command metandi [20]. As these are random effects models that may give undue weight to smaller studies, an additional sensitivity analysis was undertaken limited to studies with > 200 GS events.

The *I*^2^ statistic was used to assess heterogeneity between the sensitivity and specificity estimates in addition to visual inspection of the HSROC curves [21]. All analyses were performed using Stata version 17.

Formal testing for publication bias was undertaken by a regression of the log diagnostic odds ratio against 1/√effective sample size (ESS), weighted by ESS, with a *P* < 0.05 for the slope coefficient indicating significant asymmetry [22] (see Additional file 1, Supplemental Methods, Statistical Methods and Interpretation for details).

## Results

### Qualitative synthesis

#### Study selection

**Fig. 1:**
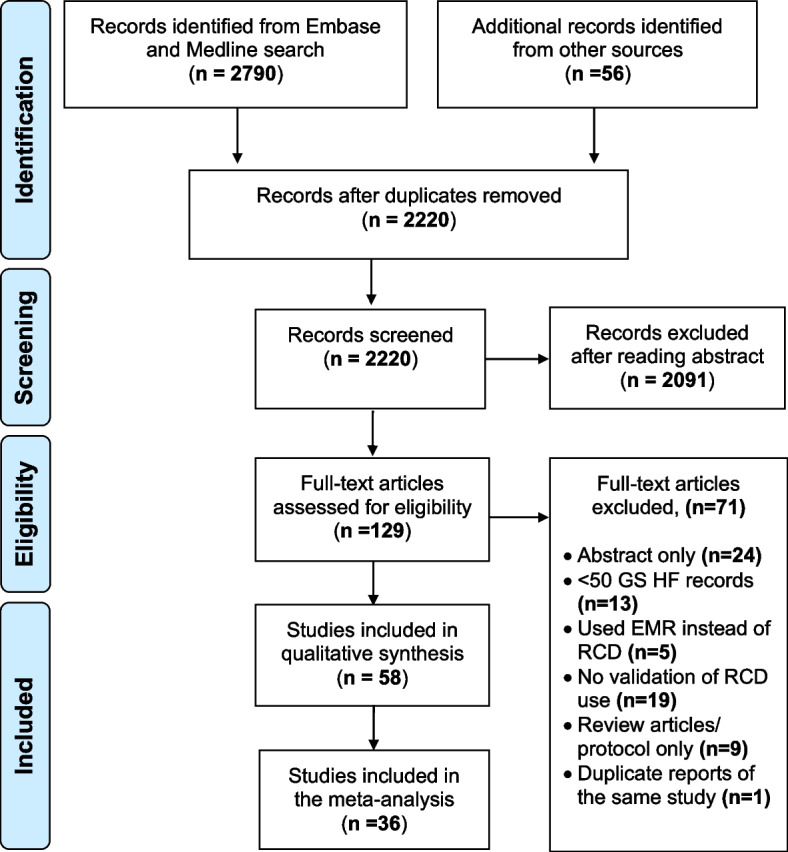
Preferred Reporting Items for Systematic Reviews and Meta-Analyses (PRISMA) flowchart summarising the study selection process. *Legend:* EMR indicates electronic medical records; GS, gold standard; HF, heart failure; n, number of records and RCD, routinely collected healthcare data

The initial Embase and MEDLINE searches yielded 2790 articles in total and an additional 56 records were identified through a manual search of references during full-text review. After the removal of duplicates and non-English language articles and abstract review, 129 articles were selected for full-text review. Of these, 71 were excluded and 58 articles were included in the final synthesis (Fig. 1).

#### Study characteristics

The 58 studies included 48,643 GS HF events in total. 34 studies (including 30,458 GS HF events) assessed acute HF outcomes [23–57], 21 studies (including 5210 HF events) assessed prevalent HF [12, 49, 58–76] while three studies (with 12,975 HF events) assessed both [77–79]. The majority of the studies (59%) were conducted in the USA and Canada. Additional file 1: Table S1 and Table S2 summarize the characteristics of the 58 studies.

#### Study quality assessment

The overall risk of bias was low for 28 (48%) studies (Additional file 1: Table S3). Of the remaining 30 studies, 7 had at least one high-risk domain and 23 had one or more domains with unclear risk of bias. Of 7 studies with high-risk domains, 6 had a reference standard at risk of not correctly classifying the target condition [28, 57, 68, 70, 71, 79] while, in one study, patients were inappropriately excluded from the analysis as they did not receive the reference standard [57]. Concerns regarding applicability were low for 42 studies (72%). Fourteen of the 16 studies with “some concern” regarding applicability were also considered “at risk” for overall risk of bias, with concerns about the reference standard being the most common issue in both areas.

#### Gold standard data sources and definition

Forty-nine (85%) studies used hospital medical records as the GS data source (Additional file 1: Table S4 summarizes the sources of routine and GS data). The remaining studies used primary care records (2 studies) [49, 76], and specialty databases or registries containing coded clinical data (5 studies) [12, 24, 35, 42, 57]. One study assessed outcomes against participant self-report [71], and another study conducted prospective medical assessments and echocardiography [37].

Most studies (85%) undertook a further adjudication step of the GS source data conducting clinical adjudication of the medical records according to study defined or guideline criteria. Three studies used the recoding of medical records by professional coders as the GS source [28, 68, 79] while the remaining six studies did not undertake any adjudication (Additional file 1: Table S5 summarizes the GS ascertainment methods used, and Table S6 the main guideline criteria used for GS adjudication).

#### Routine data sources and definition

Forty-two (72%) studies relied solely on admitted care or inpatient data sources, whilst 15 (26%) studies also used outpatient or emergency department data [23, 30, 40–42, 45, 49–51, 53, 54, 59, 71, 76, 77]. One (2%) study only used outpatient data [67]. Additional file 1: Table S4 summarises the main routine data sources. 42 studies (72%) used HDD as the main RCD source. Only three (5%) studies included prescribing data [39, 57, 71], while two (3.5%) studies included laboratory data [23, 50] 50 (86%) studies used only one RCD source, whilst eight (14%) studies used a combination of two or more sources [23, 39, 50, 57, 71–73, 76]. Two (3%) studies combined coded HDD with machine learning algorithms and keyword searches to ascertain HF events from free text HDD, electronic medical records, and discharge summaries [72, 73].

All the studies identified used data coded in one of three revisions of the ICD coding system (ICD-8, -9, or -10) with some studies using more than one. 32 (55%) studies used ICD-9 codes only, 16 (28%) studies used ICD-10 codes only and one (2%) used ICD-8 codes only [28]. Nine (16%) studies used a combination of revisions [25, 34, 39, 48, 50, 53, 65, 70, 76].

The coding algorithms used varied considerably between studies. Four (7%) studies did not define the specific coding algorithm used [25, 29, 58, 62]. The commonest ICD-9 code used was 428.x (heart failure) alone (17 studies) or in combination with other codes (20 studies). The commonest ICD-10 code used was I50.x (heart failure) alone (9 studies) or in combination with others (15 studies). Additional file 1: Tables S7 and S8 summarize the ICD-9 and -10 coding algorithms used respectively, while Additional file 1: Table S9 includes a list of all the HF codes used in the studies along with their definitions.

Most studies specified the ICD HF code position (primary, secondary, any) within the database*.* Among 37 studies ascertaining acute HF, 4 (11%) studies reported algorithms with HF codes in the primary position and any position separately [28, 30, 44, 56], 11 (30%) only reported algorithms with HF codes in the primary position, and 21 (57%) only reported algorithms with codes in any position. One study algorithm (2%) used codes in positions 1–6 [36].

### Ascertainment of acute heart failure

#### Results of individual studies

Table 1 summarizes the agreement statistics of the main study algorithm(s) for each study considering acute HF grouped by country (as RCD sources are likely to be similar) and ordered by sensitivity or PPV (highest to lowest). There was a wide range of performance across studies with sensitivities ranging from as low as 13% to > 90%. Only 8/23 (35%) studies reported a sensitivity > 80%. Although specificity also ranged widely between 20 and > 90%, 17/21 (81%) studies reported a specificity > 80%.

**Table 1:** Agreement statistics for the best ICD code-based algorithm(s) for acute heart failure studies

**First author**	**Year**	**Country**	**GS HF** **events**	**RCD source**	**GS source**	**ICD version**	**ICD algorithm/code pos.**	**Sensitivity** **(95% CI)**	**Specificity** **(95% CI)**	**PPV** **(95% CI)**	**NPV** **(95% CI)**	**Kappa** **(95% CI)**
Huang [33]	2017	USA	369	HDD	MR	9	Best alg.	96.6 (96.3–96.8)	–	92.3 (92.0–92.6)	–	–
Rosamond [56]	2012	USA	425	HDD	MR	9	Any	95.0	17.0	62.0	–	–
							1^ry^	43.0	95.0	93.0	–	–
Fisher [28]	1992	USA	788	ACD	MR (re-coded to ICD–9)	8	Any	89.0 (86.6–91.1)	95.4 (94.9–95.9)	71.0 (68.1–73.8)	98.6 (98.2–98.8)	–
			402				1^ry^	85.0 (81.2–88.4)	99.2 (99.0–99.4)	87.0 (83.3–90.2)	99.1 (98.8–99.2)	–
Birman-Deych [78]	2005	USA	–	Registry	MR	9	Any	83.0	86.0	85.0	–	–
Schellenbaum [47]	2006	USA	712	HDD	MR	9	Any	80.8 (77.7–83.6)	90.4 (89.6–91.2)	53.6 (50.6–56.7)	97.2 (96.6–97.6)	–
Heckbert [32]	2004	USA	795	HDD	MR	9	Any	79.0 (76.0–81.8)	97.7 (97.6–97.9)	45.3 (42.7–48.0)	99.5 (99.4–99.6)	0.56 (0.53–0.59)
Alqaisi [23]	2009	USA	260	ACD	MR	9	Any/Der.	76.3	74.5	–	–	–
							Any/Val.	78.5	68.6	–	–	–
Goff [31]	2000	USA	1376	Registry	MR	9	Any/alg.3	67.1 (64.5–69.6)	92.6 (91.7–93.4)	77.1 (74.6–79.5)	88.3 (87.3–89.3)	–
Psaty [44]	2016	USA	1863	ACD	MR	9	Any	53.5 (51.2–55.8)	89.9 (88.9–90.9)	72.5 (70.1–74.9)	79.6 (78.3–80.8)	–
							1^ry^	27.2 (25.2–29.3)	99.0 (98.6–99.3)	93.2 (90.7–95.2)	73.2 (72.0–74.5)	–
Cohen [50]	2020	USA	1172	ACD	MR	9, 10	Any	45.6 (42.7–48.5)	88.2 (85.8–90.3)	84.0 (80.9, 86.7)	54.4 (51.7–57.0)	–
Presley [55]	2018	USA	360	HDD	EMR	9	1^ry^	45.1 (25.1–65.1)	99.4 (99.2–99.6)	89.7 (86.8–92.7)	93.9 (89.1–98.6)	–
Allen [77]	2014	USA	46	Registry	MR	9	Alg. 2	41.7 (16.1–72.2)	98.1(96.1–99.0)	33.3 (12.8–63.1)	98.6 (96.8–99.4)	–
Jollis [35]	1993	USA	1788	ACD	Registry	9	Any	36. 0 (33.8–38.3)	96.0 (95.6–96.4)	59.2 (56.3–62.2)	90.3 (89.7–90.8)	0.39
Li [57]	2011	USA	477	ACD ^a^ + registry	Registry	9	Any	27.9 (23.9–32.1)	75.6 (72.0–79.0)	47.8 (41.8–53.9)	56.7 (53.1–60.2)	–
							Best alg	13.0 (10.1–16.4)	98.0 (96.5–99.0)	83.8 (73.4–91.3)	–	–
Roger [45]	2004	USA	658	HDD	MR	9	1^ry^	–	–	82.0 (81.1–82.9)	–	–
McCullough [41]	2002	USA	200	ACD	MR	9	Study alg	–	–	73.8	–	–
Frolova [30]	2015	Canada	733	**HDD**^**b**^	MR	10	Any	92.8 (90.6–94.5)	20.5 (12.0–31.6)	92.1 (90.0–94.0)	22.1 (12.9–33.8)	–
							1^ry^	73.9 (70.6–77.1)	71.2 (59.4–81.2)	96.3 (94.4–97.7)	21.4 (16.4–27.1)	–
Juurlink [79]	2006	Canada	482	**HDD**	MR re-coding	10	1^ry^	79.0 (75.0–83.0)	99.5 (99.4–99.6)	85.0 (82.0–89.0)	99.3 (99.1–99.4)	0.82 (0.79–0.84)
Austin [24]	2002	Canada	5475	**HDD**	Registry	9	1^ry^	58.5 (57.2–59.8)	96.8 (96.6–96.9)	65.1 (63.8–66.5)	95.8 (95.6–96.0)	0.58
Lee [38]	2005	Canada	1808	**HDD**	MR	9	1^ry^	–	–	94.3 (93.1–95.4)	–	–
Blackburn [25]	2011	Canada	345	**HDD**	MR	9	1^ry^	–	–	73.8	–	–
						10	1^ry^	–	–	84.5	–	–
Cozzolino [27]	2019	Italy	124	HDD	MR	9	1^ry^	96.0 (91.0–99.0)	90.0 (81.0–96.0)	94.0 (88.0–97.0)	93.0 (85.0–98.0)	–
Fonseca [29]	2008	Portugal	168	HDD	MR	9	Any	77.4 (70.3–83.5)	59.1 (46.3–71.0)	82.8 (76.0–88.4)	50.6 (39.0–62.2)	–
Bosco-Levy [26]	2019	France	229	HDD	MR	10	Any	64.2 (58.0–70.4)	–	60.5 (53.7–67.3)	–	–
Mahonen [39]	2013	Finland	313	Multiple^a^	MR	8, 9, 10	Study alg.	48.5 (42.9–54.2)	99.7 (99.5–99.8)	85.9 (79.7–90.5)	85.9 (79.7–90.5)	–
Merry [42]	2009	Netherlands	154	HDD	Registry	9	Any	43.0 (35.3–51.2)	99.9 (99.9–100.0)	80.0 (69.7–87.6)	99.6 (99.5–99.7)	–
Ingelsson [34]	2005	Sweden	321	HDD	MR	8, 9, 10	1^ry^	–	–	95.0 (89.6–97.8)	–	–
							Any	–	–	81.7 (76.9–85.7)	–	–
Schaufelberger [46]	2020	Sweden	911	HDD	MR	10	Any	–	–	94.4	–	–
Thygesen [54]	2011	Denmark	50	**HDD**	MR	10	1^ry^	–	–	100 (92.9–100)	–	–
Mard [40]	2010	Denmark	637	**HDD**	MR	10	Any	–	–	84.0 (81.2–86.6)	–	–
Delekta [51]	2007	Denmark	418	**HDD**	MR	10	Any	–	–	83.6 (80.1–86.7)	–	–
Kümler [37]	2008	Denmark	429	**HDD**	Study doctor^c^	10	Any	29.4 (25.1–33.9)	98.9 (98.5–99.3)	80.8 (73.7–86.6)	90.0 (88.9–91.1)	–
Sundbøll [53]	2016	Denmark	100	**HDD**	MR	8,10	1st any/1^ry^	–	–	76.0 (66.0-83.0)	–	–
Pfister [52]	2013	UK	379	HDD	MR	10	Any	–	–	95.7	–	–
Khand [36]	2005	UK	216	HDD	MR	10	1–6	–	–	86.7 (82.4–90.1)	–	–
Teng [48]	2008	Australia	1001	HDD	MR	9, 10	1^ry^	–	–	98.8	–	–
Ono [43]	2020	Japan	5404	ACD	EMR	10	1^ry^	–	–	95.7 (95.2–96.2)	–	–

*Studies grouped by country and ordered by sensitivity or PPV (highest to lowest).* Some studies contribute > 1 algorithm if assessing codes in > 1 position, or different ICD versions. See Table 1 in Additional file 1 for details of the algorithms used

*ACD* indicates administrative claims data where the data source is specified as claims data by study, *Alg.* algorithm, *CI* confidence interval, *EMR* electronic medical record, *GS* gold standard, *HDD* hospital discharge data, *HF* heart failure, *ICD* International Classification of Diseases, *MR* medical records, *Pos.* position, *PPV* positive predictive value, *NPV* negative predictive value, *RCD* routinely collected data, *Val.* validation cohort

^a^Two or more data sources

^b^RCD sources highlighted in bold from one country all used the same (or similarly structured) RCD source

^c^Prospective history and examination by study doctor

#### Meta-analysis

Sufficient data for meta-analysis was available for 17,986 GS HF events from 17/37 studies assessing RCD for acute HF. The funnel plot for publication bias with the superimposed regression line is shown in Additional file 1: Figure S2. The *p* value for the slope coefficient was not statistically significant (*P* value = 0.73) indicating a symmetrical funnel plot and a low likelihood of publication bias.

Table 2 provides the summary statistics for acute and prevalent RCD algorithms overall and according to the diagnostic position of HF codes. The summary sensitivity and specificity for acute HF studies were 63.5% (95% CI 51.3–74.1) and 96.2% (95% CI 91.5–98.3) respectively (Table 2). The agreement was similar in studies which included codes in the primary diagnostic position and any diagnostic position. When the analysis was restricted to 14 studies (17,540 GS HF events in total) with > 200 GS HF events the summary sensitivity was lower while specificity remained unchanged (Table 2 and Additional file 1: Figure S3a). When the analysis was restricted to 9 studies at low risk of bias, summary sensitivity was lower while specificity was similar (Table 2).

**Table 2:** Agreement statistics for coding algorithms ascertaining acute and prevalent heart failure according to coding position

Coding algorithms according to event type and code position	Algorithms (*N*)	Sensitivity (95% CI)	*I*^2^ for sensitivity (95% CI)	Specificity (95% CI)	*I*^2^ for specificity (95% CI)
Acute HF					
All	17	63.5% (51.3–74.1)	99.3 (99.0–99.2)	96.2% (91.5–98.3)	99.7 (99.6–99.7)
All studies with > 200 GS events	14	59.8% (48.1–70.5)	99.3 (99.1–99.4)	96.2% (92.1–98.2)	99.6 (99.6–99.7)
Studies at low risk of bias	9	55.5% (45.1–65.4)	98.9 (98.7–99.2)	97.2% (89.7–99.3)	99.7 (99.7–99.8)
Any diagnostic position	13	62.3% (47.7–75.0)	99.5 (99.4–99.6)	94.2% (84.0–98.1)	99.7 (99.7–99.8)
1^ry^ diagnostic position	7	71.0% (49.4–86.0)	99.8 (99.7–99.8)	97.8% (93.4–99.3)	99.7 (99.7–99.8)
Prevalent HF					
All	21	63.7% (55.3–71.3)	98.6 (98.3–98.8)	98.1% (97.0–98.8)	98.7 (98.5–98.9)
All studies with > 200 GS events	10	60.8% (50.9–70.6)	99.4 (99.3–99.5)	98.1% (96.4–99.0)	99.2 (99.0–99.4)
Studies at low risk of bias	8	64.3% (54.0–73.4)	98.9 (98.6–99.2)	97.7% (96.2–98.6)	97.9 (97.2–98.6)
Any diagnostic position	17	63.0% (53.9–71.3)	98.7 (98.4–98.9)	98.2% (96.9–99.0)	99.0 (98.8–99.2)
2^ry^ diagnostic position	4	66.4% (45.8–82.2)	99.2 (98.9–99.5)	97.1% (96.0–98.0)	88.9 (79.6–98.3)

*CI* indicates confidence intervals, *HF* heart failure, *I*^2^ *I*^2^ statistic describing the percentage of variation across studies that is due to heterogeneity rather than chance, *N* number of study algorithms (the same study can contribute > 1 algorithm in the subgroups if > 1 diagnostic position used, or the same study assessed acute and prevalent HF)

**Fig. 2:**
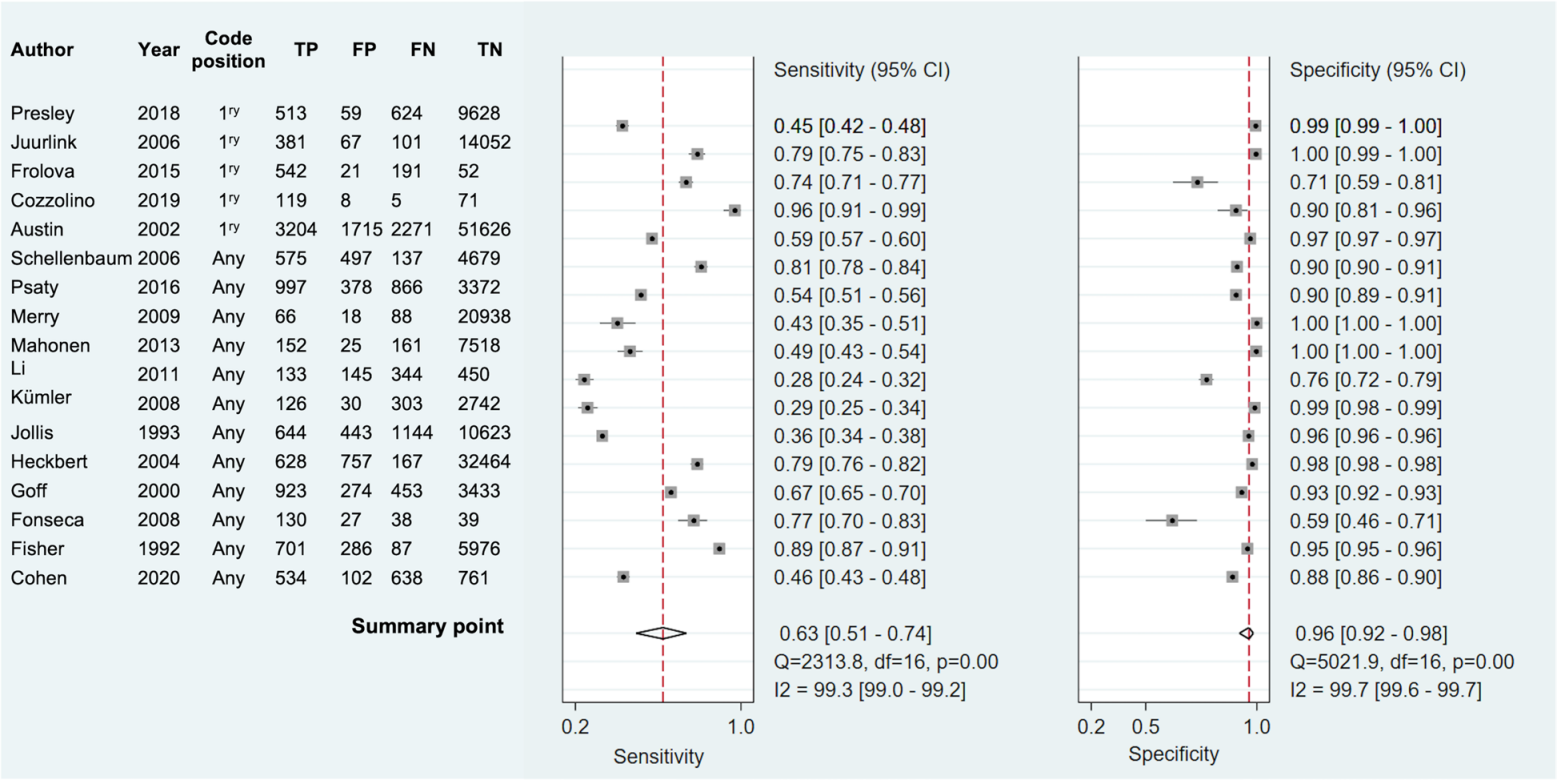
Forest plot of paired sensitivities and specificities of study algorithms ascertaining acute heart failure. *Legend*: Algorithms sorted by diagnostic code position. Summary points estimated using a bivariate random effects model. CI indicates confidence intervals; FN, false negatives; FP, false positives; I^2^, I^2^statistic describing the percentage of variation across studies that is due to heterogeneity rather than chance; TN, true negatives and TP, true positives

**Fig. 3:**
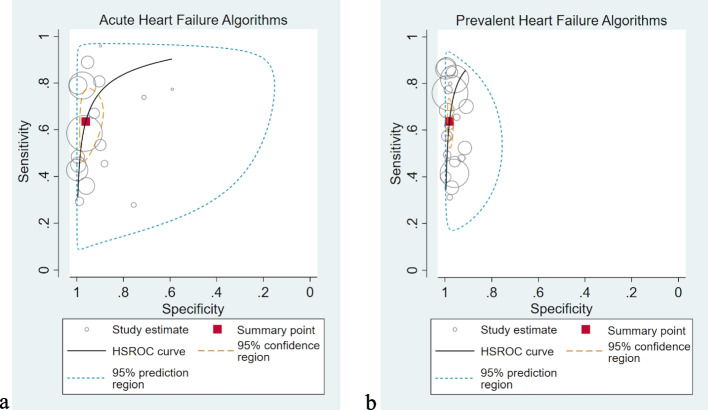
SROC plots for the diagnostic accuracy of coding algorithms ascertaining acute and prevalent heart failure. *Legend*: **a** Acute heart failure (HF) algorithms and **b** Prevalent HF algorithms. HSROC indicates hierarchical summary receiver operating characteristic curve, grey circle, the sensitivity and (1-specificity) of an individual study with the size of the circle proportionate to study size; summary point, summary sensitivity, and specificity; 95% confidence region, 95% confidence region for the summary point, and the 95% prediction region, the area in which we can say with 95% certainty the true sensitivity and specificity of a future study will be contained

Figure 2 shows the forest plot of paired sensitivities and specificities for acute HF studies. There was marked heterogeneity between studies ascertaining acute HF (*I*^2^ 99.3% and 99.7% for sensitivity and specificity respectively). The SROC plot for acute HF (Fig. 3a) has a wide 95% prediction region with individual study algorithms scattered away from the HSROC curve also suggesting considerable heterogeneity between studies, with no clear relationship between sensitivity and specificity. Heterogeneity remained regardless of the coding position used (Additional file 1: Figure S4).

### Subgroup analysis

Given the significant heterogeneity between studies, Additional file 1: Table S10 summarises agreement statistics for studies ascertaining acute HF according to other subgroups of interest that are potential sources of heterogeneity. While there were differences in summary statistics between subgroups, they had wide confidence intervals. However, algorithms from studies using medical records as the GS data source reported a higher summary sensitivity (72.6%, 95% CI 61.2–81.7) than those using registry data (41.2%, 95% CI 30.3–53.0) with similar summary specificities. Four studies with < 1500 participants had higher summary sensitivity (75.3%, 95% CI 41.4–93.0) and lower specificity (76.1%, 95% CI 63.2–85.4) compared to 13 studies with ≥ 1500 participants (59.8%, 95% CI 48.2–70.5 and 97.9%, 95% CI 95.4–99.1 respectively).

There was considerable heterogeneity with *I*^2^ ≥ 98% within all subgroups (Additional file 1: Table S10). Some of these subgroups only included a small number of studies and the summary results should be interpreted with caution.

### Ascertainment of prevalent heart failure

#### Results of individual studies

Table 3 summarizes the agreement statistics of the main study algorithm(s) for each study ascertaining prevalent HF grouped by country and ordered by sensitivity or PPV (highest to lowest).

**Table 3:** Agreement statistics for the best algorithm (s) assessing prevalent heart failure

First author	Country	GS HF events	RCD source	GS source	ICD coding		Sensitivity (95% CI)	Specificity (95% CI)	PPV (95% CI)	NPV (95% CI)	Kappa (95% CI)
					Version	Pos./Alg					
Borzecki [67]	USA	82	ACD	MR	9	Any	77.0	99.0	–	–	0.74
Birman-Deych [78]	USA	11014	Registry	MR	9	Any	76.0 (75.2–76.8)	98.0 (97.7–98.2)	97.0 (96.6–97.3)	82.4 (81.8–83.0)	–
Rector [71]	USA	218	**ACD** ^a^	Self-report	9	Best alg.	70.2 (63.6–76.2)	91.0 (90.0–91.9)	33.3 (29.0–98.4)	98.0 (97.4–98.4)	–
Allen [77]	USA	108	Registry	MR	9	Alg. 2	61.5 (39.6–79.5)	98.5 (96.6–99.3)	68.6 (44.9–85.4)	97.9 (95.9–99.0)	–
Van Doorn [75]	USA	217	HDD	MR	10	Any	56.7 (49.8–63.4)	98.7 (96.7–99.6)	96.9 (92.1–99.1)	76.3 (71.8–80.4)	0.59
Fleming [59]	USA	103	**ACD**	MR	9	Study alg.	46.6 (36.7–56.7)	95.9 (94.9–96.7)	37.2 (28.9–46.2)	97.1 (96.3–97.8)	0.38
Kieszak [69]	USA	145	**ACD**	MR	9	Any	–	–	–	–	0.38
Etzioni [12]	USA	249	HDD	Registry	9	Secondary	–	–	–	–	0.22
Schultz [76]	Canada	99	**HDD** + ACD	Primary care record	8, 9, 10	Alg. 4	84.8 (76.2–91.3)	97.0 (96.2–97.7)	55.6 (47.3–63.7)	99.3 (98.9–99.6)	–
Xu [72]	Canada	296	**HDD**	MR	10	Any	57.4 (51.8–63.0)	99.2 (98.8–99.6)	92.4 (88.6–96.2)	93.4 (92.3–94.5)	–
			+ EMR			Any + MLA	83.3 (73.9–92.8)	97.3 (95.6–98.9)	83.3 (73.9–92.8)	97.3 (95.6–98.9)	–
Juurlink [79]	Canada	1371	**HDD**	Re–coded MR	10	Secondary	82.0 (80.0–84.0)	96.0 (95.6–96.3)	68.0 (65.0–70.0)	98.1 (97.8–98.3)	0.71 (0.69–0.73)
So [65]	Canada	55	**HDD**	MR	10	Secondary	80.0 (67.0–89.6)	97.8 (93.8–99.6)	93.6 (82.5–98.7)	92.5 (86.9–96.2)	–
					9	Secondary	81.8 (68.7–90.5)	96.4 (91.8–98.8)	90.0 (78.2–96.7)	93.0 (87.5–96.6)	–
Quan (2002) [63]	Canada	128	**HDD**	MR	9	Any	77.3 (69.1–84.3)	98.7 (97.8–99.3)	87.6 (80.1–93.1)	97.3 (96.2–98.2)	0.80
Quan (2008) [70]	Canada	333	Re-coded MR	MR	9	Any	71.8 (66.6–76.5)	99.3 (99.0–99.5)	90.2 (86.0–93.5)	97.5 (96.9–98.0)	0.78
			**HDD**	MR	10	Any	68.6 (63.2–73.5)	99.3 (99.0–99.6)	90.1 (85.8–93.5)	97.2 (96.6–97.7)	0.76
Humphries [60]	Canada	58	**HDD**	MR	9	Any	65.5 (51.9–77.5)	95.0 (93.2–96.4)	50.0 (38.3–61.7)	97.3 (95.9–98.3)	0.53
Wilchesky [49]	Canada	1057	ACD	Primary care record	9	Any	41.5 (38.5–44.6)	96.1 (95.7–96.4)	44.4 (41.3–47.6)	95.6 (95.2–95.9)	–
Henderson [68]	Australia	392	HDD	Re-coded MR	10	Any (98–99)	87.0 (83.1–90.2)	99.4 (99.2–99.6)	89.9 (86.2–92.7)	99.3 (99.0–99.5)	0.88
		153				Any (00–01)	86.3 (79.7–91.0)	99.7 (99.6–99.8)	86.3 (79.7–91.0)	99.7 (99.6–99.8)	0.86
Powell [61]	Australia	172	HDD	MR	9	Secondary	62.2 (54.5–69.5)	98.3 (97.4–98.9)	81.7 (74.0–87.9)	95.4 (94.2–96.4)	–
Preen [62]	Australia	100	HDD	MR	9	Any	40.0 (30.3–50.3)	99.8 (99.5–99.9)	90.9 (78.3–97.5)	97.0 (96.1–97.7)	–
Sarfati [64]	New Zealand	64	HDD	MR	9	Any	31.3 (20.2–44.1)	98.0 (96.4–99.0)	66.7 (47.2–82.7)	91.8 (89.2–94.0)	0.38 (0.25–0.51)
Chong [58]	Singapore	469	HDD	MR	9	Secondary	35.4 (31.1–39.9)	97.1 (96.5–97.7)	65.9 (59.2–71.7)	90.6 (89.5–91.6)	0.40 (0.39–0.42)
Kaspar [73]	Germany	222	HDD	MR	10	Any + MLA	83.8 (78.3–88.4)	97.2 (95.8–98.2)	89 .0 (83.9–92.9)	95.7 (94.1–97.0)	–
			+ EMR			Any	49.5 (42.8–56.3)	99.1 (98.2–99.7)	94.0 (88.1–97.6)	87.9 (85.6–89.9)	–
Soo [66]	UK	546	HDD	MR	10	Any	52.4 (48.2–56.5)	91.6 (90.5–92.6)	56.0 (51.6–60.2)	90.4 (89.2–91.5)	0.45
Luthi [74]	Switzerland	52	HDD	MR	10	Any	48.1 (34.0–62.4)	93.2 (91.3–94.8)	30.5 (20.8–41.6)	96.7 (95.2–97.8)	0.32 (0.21–0.43)

Studies from one country using the same RCD source are shown in bold lettering

*ACD* indicates administrative claims data, *Alg.* algorithm, *CI* confidence interval, *EMR* electronic medical record, *GS *gold standard, *HDD *hospital discharge data, *HF* heart failure, *ICD* International Classification of Diseases, *MLA* machine learning algorithm, *Pos. *position, *PPV *positive predictive value, *NPV* negative predictive value, *RCD* routinely collected data

^a^ACD including pharmacy claims data

There was a wide range of performance across studies similar to acute HF studies, but a specificity ≥ 90% was reported by all 22 studies reporting specificities while only 27% reported a sensitivity ≥ 80%.

#### Meta-analysis

Twenty-one of 24 studies (including 19,840 GS HF events) ascertaining prevalent HF provided sufficient data for meta-analysis. Statistical testing for publication bias showed no significant asymmetry (*P* value = 0.57) indicating a low likelihood of publication bias (Additional file 1: Figure S2). The overall summary sensitivity and specificity were 63.7% (95% CI 55.3–71.3) and 98.1% (95% CI 97.0–98.8) respectively (Table 2). The result of restricting the analysis to 10 studies with > 200 GS events was similar to the impact on acute HF (Table 2 and Additional file 1: Figure S3b). Restricting the analysis to 8 studies at low risk of bias produced similar summary sensitivity and specificity to the overall result (Table 2).

**Fig. 4:**
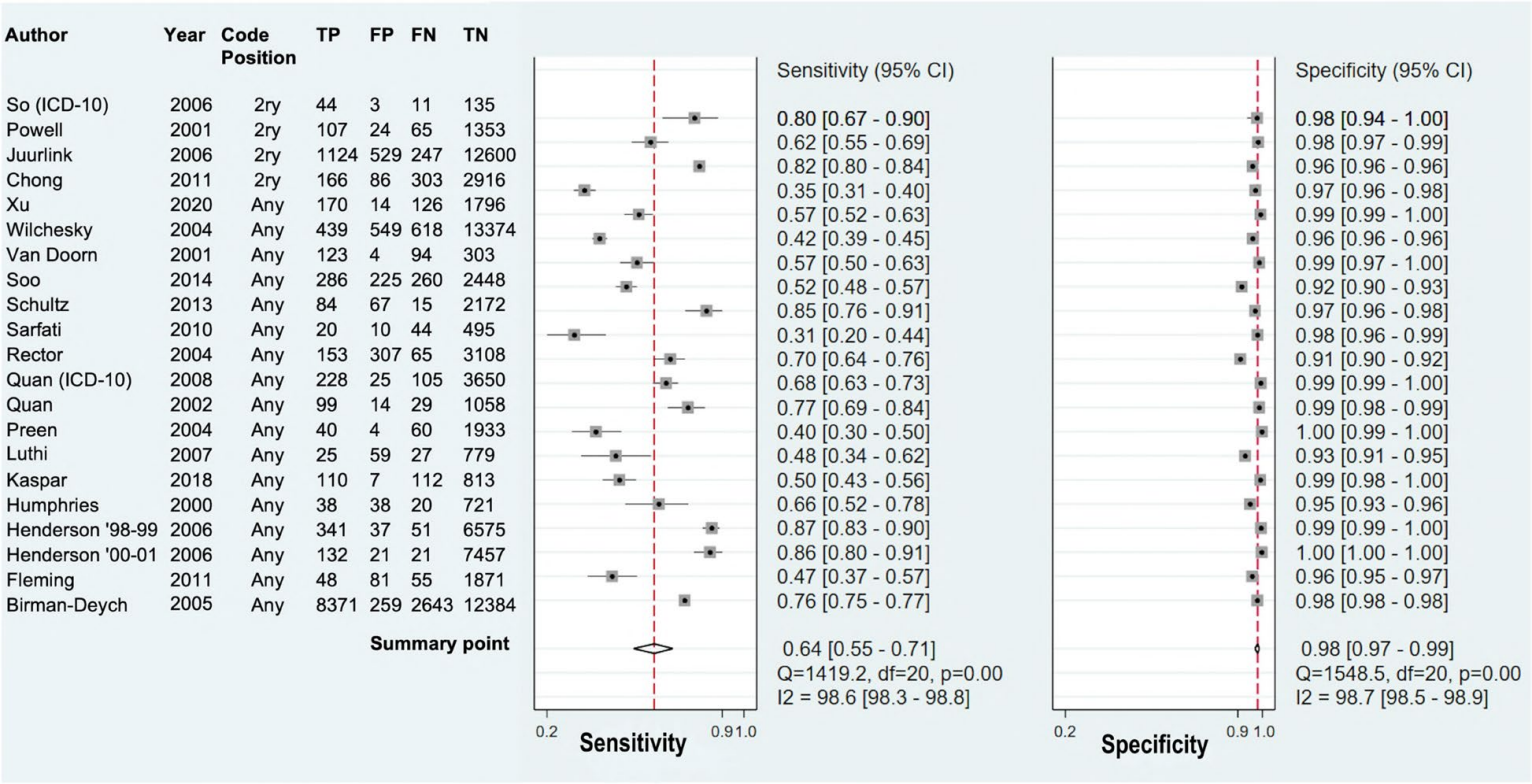
Forest plot of paired sensitivities and specificities of study algorithms ascertaining prevalent heart failure. *Legend*: Algorithms sorted by diagnostic code position. Summary points are estimated using a bivariate random effects model**. **CI indicates confidence intervals; FN, false negatives; FP, false positives; I^2^, I^2^ statistic describing the percentage of variation across studies that is due to heterogeneity rather than chance; TN, true negatives and TP, true positive

Figure 4 shows the forest plot of paired sensitivities and specificities for prevalent HF studies. There was significant heterogeneity between studies similar to acute HF studies (Table 2, Fig. 3b, Additional file 1: Figure S5).

## Discussion

RCD sources are becoming increasingly accessible to researchers and are an invaluable resource for cost-effective, streamlined clinical research. The present study demonstrated that acute HF outcomes ascertained using RCD have good specificity (96%) but lack sensitivity (63%) with similar results for prevalent HF outcomes. This indicates that whilst RCD-based ascertainment is effective at correctly identifying people who have HF, it missed one-third of cases, suggesting that further improvements are required in HF outcome ascertainment methods. The wide confidence intervals around the summary estimate of sensitivity are compatible with RCD-based ascertainment methods missing between 45 and 19% of acute heart failure cases. Furthermore, there was significant heterogeneity between studies and within subgroups which is not explained by differences in RCD coding algorithms, the GS or the country of origin, study size, or year of publication, suggesting there may be other factors such as differences in the populations studied. Therefore, both the summary statistics and subgroup analysis must be interpreted with caution.

A previous review suggested that the use of broader parameters along with laboratory and prescription data may help identify more cases [13]. However, this study has not been able to confirm this, as there were only a few studies using these sources. Eight studies used algorithms combining different sources, coding combinations, periods of data identification etc. [23, 31, 33, 39, 57, 59, 76, 77]. However, the sensitivity in these studies was no different from other studies with simpler algorithms and RCD sources, indicating that the use of complex algorithms did not necessarily improve sensitivity [23, 33, 76]. Using multiple codes from the same source compared to I50x/428x alone (broad vs narrow algorithms) has also not led to a significant increase in sensitivity for acute HF studies (67.1% vs 70.7%) in this meta-analysis (Additional file 1: Table S10). However, this comparison is again between the results of different studies. One study of 99 GS events compared several narrow versus broad coding definitions and found no difference in diagnostic accuracy [76]. Although using machine learning algorithms or keyword searches of free-text entries improved sensitivity this came at the cost of lower specificity in individual studies [72, 73].

### Characteristics of better performing algorithms

There were 5 studies with acute HF algorithms that performed above the estimated average with sensitivities > 75% while maintaining specificities > 90% [27, 28, 32, 47, 79]. However, two of these used re-coded medical records as the GS to assess coding practices [28, 79] and all of these studies were considered ‘at risk’ of bias. The use of recoded data may not be a true reflection of the actual presence or absence of disease and may explain the high concordance. In contrast, three studies using registry data as the GS source had worse sensitivities than average (Table 1). This suggests that differences in the GS may explain some of the variation between studies. The only commonalities of the remaining 3 high-performing studies were the use of ICD-9 coded inpatient HDD as the RCD source and adjudicated medical records as the GS.

Prevalent HF studies performed better with 12 studies demonstrating sensitivities > 75% while maintaining specificities > 96%. Five of these studies used RCD from Canadian hospital discharge abstract databases which are coded according to national standards [63, 65, 72, 76, 79]. One of these combined HDD with physician billing data obtaining a sensitivity and specificity of 84.8% and 97.0% respectively (Table 3) [76]. One Canadian study increased its sensitivity from 57.4% (95% CI 51.8–63.0) using an ICD-10 code search of HDD alone to 83.3% (95% CI 73.9–72.8%) by combining the code search with a machine learning algorithm of unstructured free-text entries while maintaining specificity [72]. Similar results were obtained by a German study where combining an ICD-10 code search of HDD with a machine learning algorithm of unstructured free-text improved sensitivity from 49.5% (95% CI 42.8–56.3) to 83.8% (95% CI 78.3–88.4) [73]. The study with the highest sensitivity, specificity, and kappa scores was an Australian study which again used re-coded medical records as the GS, which may explain the high concordance [68].

### Limitations of review

There are some limitations to this review. The availability of agreement statistics and information such as the coding algorithms used was variable and made direct comparison between all studies difficult. The quality of the available studies was variable with about half of studies assessed as ‘at risk’ of bias. However, restricting to studies with ‘low risk’ of bias resulted in similar summary estimates of sensitivity and specificity.

This meta-analysis utilizes the currently recommended bivariate and HSROC models which are random effects models that may give undue weight to smaller studies. However, the aim of the meta-analysis is not to present an exact summary but an overall estimate of the likely average sensitivity and specificity of using RCD for ascertainment of HF outcomes. The potential impact of using random-effects meta-analysis was assessed by doing an additional analysis limited to studies with > 200 GS events.

The comparisons between the different algorithms were limited as they were assessed in diverse study populations rather than within the same population, requiring cautious interpretation of the summary statistics and subgroup analysis. For example, a possible impact of the coding position was demonstrated in the meta-analysis results, with studies ascertaining acute HF in the primary position having better summary sensitivity and specificity than those using codes in any position (Table 2). However, four acute HF studies assessing the impact of coding position on diagnostic performance within each study all showed that using codes in the primary position reduces sensitivity and improves specificity compared to codes in any position (Table 1) [28, 30, 44, 56].

This review was also restricted to English language articles and 24 abstract-only studies were excluded. This may have led to publication bias along with any studies that may have been withheld from publication due to poor validation statistics. However, there was no statistically significant publication bias detected.

The WHO ICD-8, -9, and -10 codes do not support separate coding of HF sub-types (e.g., HF with preserved ejection fraction). Although some studies did include additional codes from the ICD-CM codes (USA) and the ICD-CA codes (Canada), this review could only assess the ascertainment of acute HF and prevalent HF irrespective of subtype. The implementation of the new WHO ICD-11 codes, which include heart failure codes capturing preserved, mid-range, and reduced ejection fraction, may allow HF subtypes to be captured in the future [80].

### Practical implications and future directions

When using acute HF outcomes to assess treatment effects in trials, a high false negative rate (low sensitivity) will have no impact on the point estimate of the overall treatment effect (provided the missing events are evenly distributed between the control arm and active arm), but it will reduce the statistical power of the trial and lead to widening of confidence intervals. In contrast, low specificity (high false positive rate) can lead to underestimation of treatment effects. Therefore, it is important to ensure that any steps taken to improve the sensitivity of HF algorithms have minimal impact on specificity. A logical way to achieve this may be to broaden the diagnostic codes used to capture HF (and/or combine more than one data source) as attempted by some studies and add a second method to maintain specificity such as a manual review of RCD records by clinicians to confirm or refute suspected events. This second method is less resource-intensive than GS adjudication of medical records and may improve diagnostic accuracy in a similar way to using machine learning algorithms on free text entries but has not been used in any of the studies reviewed [72, 73].

Finally, the considerable variation in agreement statistics between studies may be related to differences in coding practices. Therefore, any new RCD source or ascertainment method is likely to require validation prior to use for HF outcome ascertainment.

## Conclusions

While there is significant heterogeneity in studies assessing RCD-based HF outcome ascertainment, this study confirms that the presence of HF codes in RCD correctly identifies true HF but significantly underestimates events. Strategies used to improve case identification include the use of broader coding definitions, multiple data sources, and machine learning algorithms of free text data. However, these methods were not always successful and at times reduced specificity in individual studies. Therefore, methods used to improve case identification should also focus on minimizing false positives.

### Supplementary Information

**Additional file 1:** **Supplemental methods. Table S1.** Characteristics of studies ascertaining acute heart failure (ordered by country and number of gold standard events). **Table S2.** Characteristics of studies ascertaining prevalent heart failure (ordered by country and number of gold standard events). **Table S3.** QUADAS-2 study quality assessment. **Table S4.** Sources of routine and gold standard data by country or region. **Table S5.** Gold standard heart failure ascertainment methods used in the reviewed studies. **Table S6.** Guidelines used for gold standard adjudication. **Table S7.** ICD-9 coding algorithms used to define heart failure in the studies reviewed. **Table S8.** ICD-10 coding algorithms used to define heart failure in the studies reviewed. **Table S9.** List of ICD codes used across the studies and their definitions. **Table S10.** Summary diagnostic accuracy statistics for coding algorithms ascertaining acute heart failure according to subgroup. **Supplemental Figure S1.** Calculation of performance statistics. **Supplemental Figure S2.** Funnel plot for the meta-analysis of studies ascertaining acute and prevalent HF using effective sample size weighted regression tests of funnel plot asymmetry. **Supplemental Figure S3.** SROC plot for the diagnostic accuracy of coding algorithms in studies with > 200 gold standard (GS) heart failure (HF) events. **Supplemental Figure S4.** SROC plots for the diagnostics accuracy of RCD algorithms ascertaining acute heart failure according to coding position. **Supplemental Figure S5.** SROC plots for the diagnostics accuracy of RCD algorithms ascertaining prevalent heart failure according to coding position.

## Data Availability

This study brought together existing data openly available at locations cited in the reference documentation and all data generated or analyzed are included in the published article and supplementary files.

## Abbreviations

ACD: Administrative claims data
CI: Confidence intervals
GS: Gold standard
HDD: Hospital discharge data
HF: Heart failure
ICD: International Classification of Disease
NPV: Negative predictive value
PPV: Positive predictive value
RCD: Routinely collected healthcare data
(H)SROC: (Hierarchical) summary receiver operating characteristic
