# Supplementary material for: Accuracy of heart failure ascertainment using routinely collected healthcare data: a systematic review and meta-analysis
Source: Syst Rev. 2024 Mar 1;13:79. doi: 10.1186/s13643-024-02477-5 (PMC10905869; doi:10.1186/s13643-024-02477-5)
Supplement: Supplementary file 1 — Additional file 1: Supplemental methods. Table S1. Characteristics of studies ascertaining acute heart failure (ordered by country and number of gold standard events). Table S2. Characteristics of studies ascertaining prevalent heart failure (ordered by country and number of gold standard events). Table S3. QUADAS-2 study quality assessment. Table S4. Sources of routine and gold standard data by country or region. Table S5. Gold standard heart failure ascertainment methods used in the reviewed studies. Table S6. Guidelines used for gold standard adjudication. Table S7. ICD-9 coding algorithms used to define heart failure in the studies reviewed. Table S8. ICD-10 coding algorithms used to define heart failure in the studies reviewed. Table S9. List of ICD codes used across the studies and their definitions. Table S10. Summary diagnostic accuracy statistics for coding algorithms ascertaining acute heart failure according to subgroup. Supplemental Figure S1. Calculation of performance statistics. Supplemental Figure S2. Funnel plot for the meta-analysis of studies ascertaining acute and prevalent HF using effective sample size weighted regression tests of funnel plot asymmetry. Supplemental Figure S3. SROC plot for the diagnostic accuracy of coding algorithms in studies with > 200 gold standard (GS) heart failure (HF) events. Supplemental Figure S4. SROC plots for the diagnostics accuracy of RCD algorithms ascertaining acute heart failure according to coding position. Supplemental Figure S5. SROC plots for the diagnostics accuracy of RCD algorithms ascertaining prevalent heart failure according to coding position. [file 13643_2024_2477_MOESM1_ESM.docx]

# Additional File 1

Contents

[Additional File 1 1](#_Toc158719837)

[Supplemental Methods 2](#_Toc158719838)

[Definitions 2](#_Toc158719839)

[Systematic review protocol 3](#_Toc158719840)

[Review question adapted for QUADAS-2 assessment 9](#_Toc158719841)

[Statistical Methods and interpretation 10](#_Toc158719842)

[Supplemental Tables 11](#_Toc158719843)

[Table 1 Characteristics of studies ascertaining acute heart failure (ordered by country and number of gold standard events) 11](#_Toc158719844)

[Table 2 Characteristics of studies ascertaining prevalent heart failure (ordered by country and number of gold standard events) 17](#_Toc158719845)

[Table 3 QUADAS-2 study quality assessment 20](#_Toc158719846)

[Table 4 Sources of routine and gold standard data by country or region 23](#_Toc158719847)

[Table 5 Gold standard heart failure ascertainment methods used in the reviewed studies 24](#_Toc158719848)

[Table 6 Guidelines used for gold standard adjudication 25](#_Toc158719849)

[Table 7 ICD-9 coding algorithms used to define heart failure in the studies reviewed 27](#_Toc158719850)

[Table 8 ICD-10 coding algorithms used to define heart failure in the studies reviewed 28](#_Toc158719851)

[Table 9 List of ICD codes used across the studies and their definitions 29](#_Toc158719852)

[Table 10 Summary diagnostic accuracy statistics for coding algorithms ascertaining acute heart failure according to subgroup 32](#_Toc158719853)

[Supplemental Figures 33](#_Toc158719854)

[Supplemental Fig. 1 Calculation of performance statistics 33](#_Toc158719855)

[Supplemental Fig. 2 Funnel plot for the meta-analysis of studies ascertaining acute and prevalent HF using effective sample size weighted regression tests of funnel plot asymmetry 34](#_Toc158719856)

[Supplemental Fig. 3 SROC plot for the diagnostic accuracy of coding algorithms in studies with > 200 gold standard (GS) heart failure (HF) events 35](#_Toc158719857)

[Supplemental Fig. 4 SROC plots for the diagnostics accuracy of RCD algorithms ascertaining acute heart failure according to coding position 36](#_Toc158719858)

[Supplemental Fig. 5 SROC plots for the diagnostics accuracy of RCD algorithms ascertaining prevalent heart failure according to coding position 37](#_Toc158719859)

[References 38](#_Toc158719860)

## Supplemental Methods

### Definitions

#### Routinely collected data (RCD) sources:

1. Hospital administrative claims data: an umbrella term for data generated as part of the financial administration of hospitals. Includes hospital discharge data (HDD), billing/claims data, pharmacy data and laboratory data. In this paper HDD is defined as a subset of administrative claims data where the study mainly utilised coded inpatient/ outpatient HDD (e.g. discharge abstract data, patient/discharge registers). The term administrative claims data (ACD) is used to define all RCD sources where the data source is specified as claims data by the study, but the data source does not clearly fit in to the HDD category.
2. Laboratory and pharmacy or prescribing data: databases containing laboratory results (e.g. BNP levels) or prescription databases containing information regarding prescribed medications (e.g. diuretics). These were usually used in combination with ACD.
3. Cancer, disease or death registries: coded electronic databases that recorded information regarding patients with specific disease conditions such as cancer, myocardial infarctions or atrial fibrillation or centrally held national death registration databases.

### Systematic review protocol

| **Title of the review** | A systematic review of studies validating the use of routine healthcare data to ascertain heart failure outcomes against a gold standard ascertainment method. |
| --- | --- |
| **Authors:** | |
| **First reviewer** | - Dr Michelle Goonasekera, Clinical Research Fellow, Clinical Trials Service Unit, Richard Doll Building, Nuffield Department of Population Health, University of Oxford, Old Road Campus, Roosevelt Drive, Oxford, OX3 7LF, UK.   Email: [michelle.goonasekera@ndph.ox.ac.uk](mailto:michelle.goonasekera@ndph.ox.ac.uk)  This work is part of a project undertaken by the first reviewer in fulfilment of the academic requirements of the Doctor Medicine degree at Warwick Medical School, University of Warwick. |
| **Supervisors** | - Dr Marion Mafham, Senior Clinical Research Fellow and Consultant Nephrologist, Clinical Trials Service Unit, Nuffield Department of Population Health, University of Oxford.   Email: [marion.mafham@ndph.ox.ac.uk](mailto:marion.mafham@ndph.ox.ac.uk)   - Prof Richard Haynes, Professor of Renal Medicine and Clinical Trials, Clinical Trials Service Unit, Nuffield Department of Population Health, University of Oxford.   Email: [richard.haynes@ndph.ox.ac.uk](mailto:richard.haynes@ndph.ox.ac.uk) |
| **Support:** | |
| **Sources** | Not applicable |
| **Sponsor** | Not applicable |
| 1. **Introduction:** | |
| **Background and Rationale**  Heart failure (HF) is an important cause of morbidity and mortality in the general population. It affects 1-2% of the adult population and is a significant burden on health care, accounting for 2% of all health-care expenditure. ^1^ ^2^ Therefore, HF is an important target for treatment, requiring large randomised, controlled trials (RCT) to assess potential interventions. Such large trials can be complex and costly.^3^ For example, ascertainment of a HF admission in a clinical trial involves an arduous process of conducting clinic visits to identify potential events and gathering large numbers of clinical documents for reported events, followed by clinical adjudication. This process could be streamlined to reduce the complexity and overall cost of trials.^4-6^ Using routinely collected healthcare data to ascertain outcomes may help to achieve this goal by allowing the conduct of streamlined registry trials and low-cost, long-term trial follow-up.^7^  HF can be a problematic condition to identify with certainty even with clinical adjudication. For example, in clinical practice there is significant overlap between patients presenting with fluid overload secondary to chronic kidney disease (CKD) and those presenting with underlying cardiac dysfunction. A diagnosis in such situations requires objective evidence of structural and functional cardiac abnormality.^8^ Therefore, it may be challenging to accurately ascertain HF events purely based on routine healthcare data. A systematic review and meta-analysis of studies published up to November 2010, by McCormick et al., reports the validity of diagnostic codes for identifying HF in administrative data.^9^ Their meta-analysis of 11 studies reporting sensitivity and specificity values, showed that pooled sensitivity was 75% (95% confidence interval [CI]: 74.7–75.9) and pooled specificity was 97% (95% CI: 96.8–96.9). These findings were similar to those from two other previous reviews.^10, 11^ However, there were a limited number of studies in this review, mostly from Canada and the USA, and some studies had very small numbers of HF events. It is also possible that coding practices have improved over the last decade. Therefore, more recent evidence for using routine data (RD) for HF outcome ascertainment needs review. A more recent review has been published by Davidson et al. but it focused entirely on Europe and included electronic health records (EHR) and primary care data. ^12^ They reported sensitivities ≤66% and specificities ≥95% in most of the studies. | |
| **Objective:**  The main objective of this work is to systematically review studies validating the use of RD-based methods of HF outcome ascertainment against a gold standard method in order to summarise the available evidence supporting the use of RD in cardiovascular outcomes trials. | |
| 1. **Methods:** | |
| **Eligibility criteria** | |
| **Population:** | Studies including individuals with heart failure. No other restrictions on study population. |
| **Exposure:** | A HF diagnosis captured in a routine healthcare database using International Classification of Diseases (ICD) diagnostic codes for HF (versions 8, 9 or 10). |
| **Comparison:** | Absence of a coded HF diagnosis in the routine healthcare database of interest. |
| **Outcome of interest:** | A diagnosis of HF made using a gold standard ascertainment method (reference standard). |
| **Setting:** | Hospital admissions or ambulatory care attendance (outpatient or emergency department attendances) |
| **Study design:** | To be included in the review a study was required to have:  a) validated the use of RD for ascertainment of HF against defined gold standard (GS) ascertainment criteria.  b) at least one validation statistic (sensitivity, specificity, positive predictive value, negative predictive value, kappa statistic) or sufficient raw data to calculate them)  c) at least 50 HF events ascertained using the gold standard ascertainment method.  There were no other restrictions placed on study design. Most studies are likely to be observational based on the research question. |
| **Exclusion criteria** | |
| - Studies not using RD as the source of HF outcomes. - Studies not validating the use of RD for outcome ascertainment, - Studies using electronic medical records (EMR) as the sole RD source as EMRs are usually considered a GS data source. - Studies with <50 GS adjudicated HF events. - Studies not published in English. | |
| **Search methods** | |
| **Electronic databases** | Medline (1946 to May 2021)  Embase (1974 to May 2021) |
| **Search software** | The search will be conducted using the OvidSP platform. The planned full search strategy is included in **Appendix 1**. The initial search will be run by the first reviewer (MG).  Endnote X9 was used to manage the bibliography. |
| **Other methods used for identifying relevant research** | Hand searching of all references of full-text articles reviewed.  Use of Similar articles feature on Pubmed and Citing articles features on other platforms to find other relevant articles. |
| **Handling abstracts** | We will not be contacting the authors of abstract only publications. |
| **Methods of review** | |
| **Details of reviewers** | The main reviewer (MG) will screen all the search results based on the pre-specified inclusion and exclusion criteria. Any uncertainties will be resolved in discussion with MM and RH. All three authors will decide on the data to be extracted at the outset. MG will complete the initial data extraction and discuss any uncertainties with MM and RH. The second reviewer (MM) will verify the extracted data. |
| **Quality assessment** | The protocol will define the method of literature appraisal. No specific tool was used in the quality assessment. |
| **Data extraction** | Data will be extracted by MG into a data collection form using Microsoft Excel. Endnote X9 will be used to track relevant references. |
| **Narrative synthesis** | - The selected studies will be reviewed to summarise the routine and GS data sources, event types (inpatient, outpatient etc.), codes/ coding algorithms (including coding position) and validation statistics used. - Studies will be reviewed under two main categories:  1. Studies assessing acute HF (hospital attendance for new or worsening HF) 2. Studies assessing prevalent HF (presence pre-existing HF with or without new diagnoses of HF)  - Further subgroups of interest will include country or region, coding position (primary, secondary or any), ICD version along with routine and GS data sources and ascertainment methods used. |
| **Meta-analysis** | A meta-analysis of the extracted data is planned to assess heterogeneity between studies and to explore the reasons for heterogeneity. Coupled Forest plots will be used to explore the differences between studies assessing acute and prevalent HF and other relevant subgroups based on the data extracted. The I^2^ statistic will also be estimated as a measure of heterogeneity.  Summary receiver operating characteristic curves (SROC), summary sensitivity, specificity and diagnostic odds ratios for the main subgroups will also be assessed where sufficient data is available. All analyses will be conducted using Stata SE version 16.1 |
| **Grading evidence** | N/A |
| **3. Presentation of results** | |
| **Additional material** | Flow chart of the process, protocol, summary tables describing RD and GS data sources, summary tables of extracted validation statistics, Forest plots of studies included in the final review. |
| **Outputs from review** | - Paper in high quality cardiovascular journal. - Report and presentation to University of Warwick MD review panel - Inclusion in MD thesis. |
| **4. Timeline for review** | |
| **Protocol** | 1 month |
| **Literature search** | 2 months |
| **Data extraction** | 2 months |
| **Synthesis** | 3 months |
| **Writing up** | 2 months |

**References**

1. McMurray JJ and Pfeffer MA. Heart failure. *Lancet*. 2005;365:1877-89.

2. James SL, Abate D, Abate KH, Abay SM, Abbafati C, Abbasi N, Abbastabar H, Abd-Allah F, Abdela J, Abdelalim A, et al. Global, regional, and national incidence, prevalence, and years lived with disability for 354 diseases and injuries for 195 countries and territories, 1990-2017: a systematic analysis for the Global Burden of Disease Study 2017. *The Lancet*. 2018;392:1789-1858.

3. Sertkaya A, Wong HH, Jessup A and Beleche T. Key cost drivers of pharmaceutical clinical trials in the United States. *Clin Trials*. 2016;13:117-26.

4. Zannad F, Pfeffer MA, Bhatt DL, Bonds DE, Borer JS, Calvo-Rojas G, Fiore L, Lund LH, Madigan D, Maggioni AP, et al. Streamlining cardiovascular clinical trials to improve efficiency and generalisability. *Heart*. 2017;103:1156.

5. Calvo G, McMurray JJV, Granger CB, Alonso-García Á, Armstrong P, Flather M, Gómez-Outes A, Pocock S, Stockbridge N, Svensson A, et al. Large streamlined trials in cardiovascular disease. *European Heart Journal*. 2014;35:544-548.

6. Collins R. Back to the future: the urgent need to re-introduce streamlined trials. *European Heart Journal Supplements*. 2018;20:C14-C17.

7. Van Staa T-P, Goldacre B, Gulliford M, Cassell J, Pirmohamed M, Taweel A, Delaney B and Smeeth L. Pragmatic randomised trials using routine electronic health records: putting them to the test. *Bmj*. 2012;344:e55.

8. Segall L, Nistor I and Covic A. Heart failure in patients with chronic kidney disease: a systematic integrative review. *BioMed research international*. 2014;2014:937398-937398.

9. McCormick N, Lacaille D, Bhole V and Avina-Zubieta JA. Validity of heart failure diagnoses in administrative databases: A systematic review and meta-analysis. *PLoS ONE*. 2014;9.

10. Quach S, Blais C and Quan H. Administrative data have high variation in validity for recording heart failure. *Canadian Journal of Cardiology*. 2010;26:e306-e312.

11. Saczynski JS, Andrade SE, Harrold LR, Tjia J, Cutrona SL, Dodd KS, Goldberg RJ and Gurwitz JH. A systematic review of validated methods for identifying heart failure using administrative data. *Pharmacoepidemiology and drug safety*. 2012;21 Suppl 1:129-140.

12. Davidson J, Banerjee A, Muzambi R, Smeeth L and Warren-Gash C. Validity of Acute Cardiovascular Outcome Diagnoses Recorded in European Electronic Health Records: A Systematic Review. *Clinical epidemiology*. 2020;12:1095-1111.

**Appendix 1:**

**Medline search strategy (20.05.2021)**

|  | Search |
| --- | --- |
| 1 | (administrative data*: or administrative healthcare data: or administrative health care data: or administrative health data:). ti,ab. |
| 2 | (routine data*: or routine healthcare data: or routine health care data:). ti,ab. |
| 3 | (utilization data: or utilisation data: or claims data: or managed care data: or physician billing data: or hospitalization data: or billing data:). ti, ab. |
| 4 | (HES: or Hospital Episode Statistics:).ti, ab. |
| 5 | Validation Studies/ |
| 6 | Validation Studies as Topic/ |
| 7 | Valid*.ti,ab. |
| 8 | (outcome*: or outcome validation: or outcome assessment: outcome ascertainment:). ti,ab. |
| 9 | (adjudicate*: or outcome adjudication: or endpoint adjudication: or event adjudication:). ti,ab. |
| 10 | exp "Outcome Assessment (Health Care)"/ |
| 11 | exp Heart Failure/ |
| 12 | exp congestive heart failure/ |
| 13 | (congestive heart failure: or congestive cardiac failure:). ti,ab. |
| 14 | 1 or 2 or 3 or 4 |
| 15 | 5 or 6 or 7 or 8 or 9 or 10 |
| 16 | 11 or 12 or 13 |
| 17 | 15 and 16 |
| 18 | 14 and 17 |

**Embase search strategy (20.05.2021)**

|  | Search |
| --- | --- |
| 1 | (administrative data*: or administrative healthcare data: or administrative health care data: or administrative health data:). ti,ab. |
| 2 | (routine data*: or routine healthcare data: or routine health care data:). ti,ab. |
| 3 | (utilization data: or utilisation data: or claims data: or managed care data: or physician billing data: or hospitalization data: or billing data:). ti,ab. |
| 4 | (HES: or Hospital Episode Statistics:).ti, ab. |
| 5 | Validation Studies/ |
| 6 | Validation Studies as Topic/ |
| 7 | Valid*.ti,ab. |
| 8 | (outcome*: or outcome validation: or outcome assessment: outcome ascertainment:). ti,ab. |
| 9 | (adjudicat*: or outcome adjudication: or endpoint adjudication: or event adjudication:). ti,ab. |
| 10 | exp "Outcome Assessment (Health Care)"/ |
| 11 | exp Heart Failure/ |
| 12 | exp congestive heart failure/ |
| 13 | (congestive heart failure: or congestive cardiac failure:). ti,ab. |
| 14 | 1 or 2 or 3 or 4 |
| 15 | 5 or 6 or 7 or 8 or 9 or 10 |
| 16 | 11 or 12 or 13 |
| 17 | 15 and 16 |
| 18 | 14 and 17 |

### Review question adapted for QUADAS-2 assessment

**Review question:**

A review of studies assessing ICD coded RCD based methods of acute or prevalent HF ascertainment against a defined gold standard ascertainment method to assess the utility of RCD based ascertainment methods

**Patients:**

Any patient population with or at risk of HF

**Index test(s):**

Event identification using ICD coded routinely collected healthcare datasets. The search must involve the use of an ICD code search as the sole method or in combination with other search parameters. The study may be a primary diagnostic accuracy study (with only one test method compared to the GS) or a comparative diagnostic accuracy test (several test methods compared to the GS). In a comparative diagnostic accuracy study the index test will be the best algorithm using an ICD code search of the RCD so that the methods that are being meta-analysed are comparable.

**Reference standard**:

A study defined manual process of event identification such as medical records review

**Target conditions:**

1. Acute HF (hospital attendance for decompensated HF)

2. Hospitalisations for prevalent HF (new and pre-existing HF or HF as a co-morbidity)

### Statistical Methods and interpretation

All agreement statistics and confidence intervals (CI), were calculated using the Stata diagti command where sufficient data was available.[1] Where there were minor discrepancies between the calculated and original 95% CI, the calculated values have been reported for consistency.

Metandi, the Stata command used for meta-analysis, utilises bivariate (random effects model) and HSROC models (mixed effects model) which are the recommended models for meta-analysis of diagnostic accuracy studies. [2-4] As random effects models may give undue weight to smaller studies an additional analysis was undertaken limiting the analysis to studies with >200 GS events.

The Stata command midas was used to generate forest plots and calculate I^2^ statistics to assess heterogeneity between studies.[5] Usually the I^2^ does not account for heterogeneity explained by differences such as positivity threshold effects in diagnostic accuracy studies. Numerical estimates of heterogeneity are available within the hierarchical models used in metandi but are not easily interpreted as they are expressed using log odds scales. Therefore, visual inspection of the scatter of studies from the summary HSROC curve is considered a better depiction of the extent of observed heterogeneity. The wider the 95% prediction region, the more heterogeneity there is between studies [4].

Midas was also used generate a funnel plot for assessing publication bias with formal testing undertaken by regression of log diagnostic odds ratio (lnDOR) against the 1/√effective sample size (ESS). The DOR is considered a better summary of test accuracy than odds ratios or risk ratios while, 1/√ESS is considered a better estimate of sample size effects than the standard error of the lnDOR. ESS = (4*n*_1_*n*_2_)/(*n*_1_ + *n*_2_) where *n*_1_ is the number in the non-diseased group and *n*_2_ the number in the diseased group. [6]

## Supplemental Tables

Table 1 Characteristics of studies ascertaining acute heart failure (ordered by country and number of gold standard events)

| First author,  Year | Country | Year(s) of data collection | Sample size (GS events) | Population | RCD source (s) | RCD type | Diagnostic codes used | Coding position/ algorithms used (best algorithm in bold) | Gold standard |
| --- | --- | --- | --- | --- | --- | --- | --- | --- | --- |
| Birman-Deych  2005 [7] | USA | 1998-1999 | 23,657  **(11,014)** | Adults with atrial fibrillation aged 20-105 years | National Atrial Fibrillation registry data | ICD-9 (CM)  IP | 428.x, 398.91, 402.x1, 404x1, 404.x3 | 1. Any position (prevalent)  2. Any position (acute), at baseline hospitalisation | MRR for previous or current HF |
| Psaty  2016 [8] | USA | 1989-1993 | 5613 **(1863)** | Adults ≥ 65 years of age | ACD | ICD-9 IP | 428, 402x1, 404.x1, 404.x3 | 1. Primary position  2. Any position | MRR (CHS ^a^ adjudication criteria) |
| Jollis  1993 [9] | USA | 1985-1990 | 12,937  **(1,788)** | Patients undergoing cardiac catheterization | ACD | ICD-9 (CM) IP | 398.91, 402.x1, 428.0, 428.1, 428.9 | Any position | Cardiovascular disease database review. NYHA 1 or greater HF |
| Goff  2000 [10] | USA –  Texas | 1988-1994 | 5,083  **(1,376)** | All residents aged 25-74 hospitalized for coronary heart disease | Corpus Christi Heart Project surveillance program | ICD-9 (CM) IP | 398.91, 402.x1, 404.x, 415.0 416.9, 425.4, 428.x, 429.4, 514, 518.4, 786.0 | Any position, 3 algorithms.  1. Presence of 428  2. Presence of 428 or 402  **3. Presence of any CHF code** | MRR for a diagnosis of acute CHF, or pulmonary oedema on chest radiograph |
| Cohen  2020 [11] | USA | 2007-2015 | 2035  **(1172)** | Adults >20 years with incident HF + ejection fraction measurement | ACD +/- laboratory data | ICD-9, 10 IP/OP | 428.3X, I50.3x in any position in people with at least one 428/ I50 code in the past. | Any position. One IP or 2 OP diagnosis of 428.3x or I50.3X (diastolic HF).  16 algorithms. | MRR using Framingham criteria + ejection fraction ≥50% |
| Heckbert  2004 [12] | USA | 1994-2000 | 34,016  **(795)** | Postmenopausal women (WHI^a^ trials) | Local HDD | ICD-9 IP | 428, 425 | Any position | MR and death certificate review using WHI ^a^ criteria. |
| Fisher  1992 [13] | USA | 1984-1985 | 7,050  **(788)** | Hospitalised Medicare beneficiaries | ACD | ICD-8  IP | ICD-8: Not defined.  ICD-9: 402.x1, 428x | 1. Any position  2. Primary position | ICD-9 recoding of MR by coders blind to original ICD-8 coding |
| Schellenbaum  2006 [14] | USA | 1989-1993 | 5,888  **(712)** | Adults ≥ 65 years old | Local HDD | ICD-9 IP | 428, 425, 398.91, 402.x, 997.1. | Any position (up to 10 coding positions) | MRR - CHS ^a^ adjudication criteria |
| Roger  2004 [15] | USA - Minnesota | 1979-2000 | 7,298  **(658)** | Patients with HF | Regional HDD | ICD-9 (CM) IP/OP | 428 | Primary position | MRR - Framingham ^a^ criteria |
| Li  2011 [16] | USA | 1996-2006 | 1072  **(477)** | Patients aged ≥65 years hospitalised with HF/ myocardial infarction with an ejection fraction result on record | ACD, national disease registry and pharmacy dispensing data | ICD-9 IP | 1.428.2X +/- 428.4X  2. 428.XX  3. Absence of 427.3X in patients with digoxin prescriptions | **1. 428.xx in** **any position + ACEI/ARB prescription within 30 days**  2. Preferred definition: No IP diagnosis of atrial fibrillation/ flutter during, and >1 digoxin prescriptions 180 days before/ 30 days after the index admission. (13 definitions in total for systolic dysfunction.) | Ejection fraction <45% in registry data |
| Rosamond  2012 [17] | USA | 2005 | 705  **(425)** | Residents aged  ≥ 55 years with a RCD HF code | Regional HDD | ICD-9 (CM) IP | 428 (more extended algorithm used to screen participant) | 1. Primary position  2. Any position | MRR by physicians -definite/possible acute HF, chronic HF, not HF or unclassifiable. |
| Huang  2017 [18] | USA - Minneapolis | 2009-2014 | 447  **(369)** | Hospitalised patients with HF | Local HDD | ICD-9 IP | 428.x or 428.x excluding chronic HF codes (428.22, 428.32 & 428.42) +/- 402.x1, 404.x1, 415, 518.4 | Primary diagnostic position, 4 algorithms:   - 1. 428.x   2. 428.x + 415 + 518.4   3. 428.x + 402.x1 + 404.x1 + 415 + 518.4   4. **(428.x - chronic HF codes) + 402.x1, 404.x1, 415, 518.4** | MRR using 2014 ACC/AHA ^a^ guideline (probably HF/ probably not HF/ unclassifiable) |
| Presley  2018 [19] | USA- Tennessee | 2001-2012 | 497  **(360)** | Hospitalised patients >18 years old with diabetes | Regional HDD | ICD-9  IP or DRG codes | ICD-9: 425.x, 428.x, 404.x1, 404.x3, 398.91, 402.x1; and/or DRG code: 127 (before 2008) or 291–293 (after 2008) | Primary position (ICD-9) and DRG code. | Electronic MRR for signs and symptoms of HF (Framingham criteria ^a^) within 24 hours of admission. |
| Alqaisi  2009 [20] | USA -Michigan | 2004-2005 | 400  **(260)** | Adults (aged  ≥18 years) with a HF encounter code | ACD +/- laboratory data | ICD-9 IP/OP | 428.xx, 398.91, 402.x1 | Sixteen claims and BNP based algorithms. Best algorithm**: ≥2 OP or any IP discharge diagnosis of HF or BNP ≥200pg/ml. (Derivation and validation cohorts)** | MRR (Framingham ^a^ criteria) |
| McCullough 2002 [21] | USA | 1989-1999 | 271  **(200)** | Hospitalised patients | ACD | ICD-9 (CM) IP/OP | 428.xx, 398.91, 402.x1, 404.00, 404.x1, 404.x3, 404.10, 404.9 | At least 2 OP encounters or 1 hospitalisation coded for CHF (primary position) | MRR (Framingham^a^ or NHANES 1 criteria) |
| Allen  2014 [22] | USA | 1999-2007 | 400  **(108)** | Women with incident invasive breast cancer | Cancer registry network data | ICD-9(CM)  IP/ OP | 398.91, 402.x1, 402.x3, 404.x1, 404.x3, 422.90, 425.4, 425.9, 428.x, 425  (HF and cardiomyopathy) | 1: ≥1 primary diagnosis  **2: ≥1 primary diagnosis or ≥3 secondary diagnoses or ≥3 ED or ≥2 OP or ≥1 OP and ≥2 secondary diagnoses**  3: Any ICD-9 code for HF/cardiomyopathy  Acute and prevalent HF assessed separately. | 1. MRR for cardiologist diagnosis (ESC ^a^ criteria) OR,  2. Ejection fraction < 50% (ACC/AHA criteria) |
| Austin  2002 [23] | Canada - Ontario | 1996-2000 | 58,816  **(5,475)** | Adults (aged ≥20 years) admitted to Coronary Care Unit | National HDD | ICD-9 IP | 428 | Primary diagnostic position | Prospective diagnosis recorded during admission (Fastrak II Registry). |
| Juurlink  2006 [24] | Canada -Ontario | 2002-2004 | 14,500  **(1,853)** | Hospitalised patients | National HDD | ICD-10 (CA) IP | I50 | Most responsible diagnosis code or a secondary diagnosis (pre- or post-admission co-morbidity) | MR recoded by trained abstracters |
| Lee  2005 [25] | Canada - Ontario | 1997-1999 | 1,808  **(1,808)** | Residents (<105 years) hospitalised with HF | National HDD | ICD-9 IP | 428.x | Primary diagnostic position | MRR using Framingham ^a^ criteria |
| Frolova  2015 [26] | Canada – Alberta | 2009-2012 | 897  **(733)** | Patients hospitalised for acute HF | ED AD  Local HDD | ICD-10 IP/ED | I50.x | **HDD** or ED administrative data  1. **Primary position**  2. Any position | MRR using Carlson ^a^ criteria +/- BNP levels. |
| Blackburn  2011 [27] | Canada | 1994-2003 | 466  **(345)** | Patients eligible for prescription drug benefits hospitalised for first HF event | National HDD | ICD-9, -10 (CA) IP | Not defined | Primary position | MRR using Framingham ^a^ criteria |
| Schaufelberger  2020 [28] | Sweden | 2000-2012 | 965  **(911)** | Hospitalised patients aged  20-100 years | National inpatient register | ICD-10 IP | I50 | Any position | MRR (ESC ^a^ criteria)- definite, probable and miscoded HF |
| Mard  2010 [29] | Denmark | 2005-2007 | 758  **(637)** | Patients hospitalised with a HF diagnosis | National patient register | ICD-10 IP/OP | I11.0, I13.0, I13.2, I42.0, I42.6–9, I50.0–I50.9 | Any position | MRR (ESC ^a^ criteria) - definite HF or no HF |
| Delekta  2018 [30] | Denmark | 2007 | 500  **(418)** | Patients hospitalised with  a RCD HF diagnosis | National patient register | ICD-10 IP/OP/ED | I50.0-I50.9 | Any position | MRR (ESC ^a^ criteria) -definite, probable or non-verified acute HF |
| Pfister  2013 [31] | UK-  Norfolk | 1997-2009 | 396  **(379)** | Residents of Norfolk | National HDD | ICD-10 IP | I50 | Any position | MRR (ESC ^a^ criteria) – definite, probable, possible or no HF. |
| Ingelsson  2005 [32] | Sweden | 1970-2001 | 2,322  **(321)** | Men aged 50 years (from  1970-74) | National hospital discharge register | ICD-8, -9, -10 IP | ICD-8: 427.00, 427.10, 428.99  ICD-9: 428  ICD-10: I50, I11.0 | ICD-8, -9 or -10 codes in primary or any position | MRR (ESC ^a^ criteria) - definite, questionable or miscoded HF |
| Mahonen  2013 [33] | Finland | 1997-2008 | 7856  **(313)** | Patients from 6 geographic areas | HDD, cause of death register, drug reimbursement and pharmacy data | ICD-8, -9, -10 IP | ICD-8: 427.00, 427.10, 428 ICD-9: 402.9B, 414.8, 428 ICD-10: I50, I11.0, I13.0, I13.2 | HF code in any position in discharge register or as a cause of death, reimbursement for HF drugs and pharmacy data on the regular use of furosemide | MRR and BNP/ pro-NT-BNP review for probable, possible and no HF |
| Kümler  2008 [34] | Denmark | 1998 -1999 | 3,201  **(429)** | Hospitalised adults aged >40 years | National patient registry | ICD-10 IP | I50.0–I50.9 | Primary or secondary position | Prospective history and examination by one study physician and echocardiogram by another. (ESC ^a^ criteria) |
| Bosco-Levy  2019 [35] | France | 2014 | 429  **(229)** | Adults (≥40 years) hospitalised with electronic MR or coded HDD diagnosis of HF | National HDD | ICD-10 IP | I50.x | Any position | MRR (ESC ^a^ criteria) - definite, potential or miscoded HF |
| Khand  2005 [36] | Scotland - Glasgow | 1997-1998 | 339  **(216)** | Hospitalised patients | Local HDD | ICD-10 IP | I50.1, I50.2, I11.0, I11.1, I25.5, I42.9 | Diagnostic positions 1-6 | MRR using ESC ^a^ criteria (definite, probable and possible HF or not HF) |
| Fonseca  2008 [37] | Portugal - Lisbon | 2001 | 234  **(168)** | Patients hospitalised under Medicine | Local HDD | ICD-9 IP | Not defined | Any position | MRR for evidence of HF |
| Merry  2009 [38] | Netherlands -Maastricht | 1987-2003 | 21,110  **(154)** | Residents aged 20-59 years | Hospital discharge register | ICD-9 IP/OP | 428 | Any position | Cardiology database review. |
| Cozzolino  2019 [39] | Italy - Umbria | 2012-2014 | 458  **(124)** | Hospitalised residents ≥ 18 years | Regional HDD | ICD-9 (CM) IP | 428.x | Primary position | MRR (ESC ^a^ criteria). |
| Sundbøll  2016 [40] | Denmark | 2010-2012 | 2153  **(100)** | Patients hospitalised under medical specialties | National patient registry | ICD-8, -10 IP/OP | ICD-8: 427.09, 427.10, 427.11, 427.19, 428.99, 782.49. ICD-10: I50x, I11.0, I13.0, I13.2, I42.0, I42.6-I42.9 | Primary position or first-time secondary diagnosis of HF | MRR by physicians using study specific criteria |
| Thygesen  2011 [41] | Denmark | 1998-2007 | 950  (**50)** | Hospitalisations with a primary diagnosis of a Charlson condition [42] | National patient registry | ICD-10 IP/OP/ED | I50, I11.0, I13.0, I13.2 | Primary position | MRR by physicians using study specific criteria |
| Teng  2008 [43] | Western Australia | 1996-2006 | 1,006  **(1,001)** | Patients with a principal diagnosis of HF | Regional HDD | ICD-9, -10 IP | ICD9: 428x, 402.x1, 404.1, 404.3, 425x, 518.4, 514, 391.8, 398.91. ICD10: I50x, I11.0, I13.0, I13.2, I42x, J81, I01.8, I02.0 | ICD-9 or -10 codes in primary position | MRR (Carlson criteria ^a^ ) - possible and definite HF |
| Ono  2020 [44] | Japan | 2006-2018 | 312,213  **(5,404)** | Hospitalised patients with a type 2 diabetes code | National Hospital ACD | ICD-10 IP | I50, I11.0, I13.0, I13.2 | Primary position | ICD-10 coded physician diagnosis of HF within linked EMR system |

ACC/AHA criteria indicates American College of Cardiology/American Heart Association criteria; ACD, administrative claims data; ACEI/ ARB, angiotensin converting enzyme inhibitor/ angiotensin receptor blocker; AM, Australian modification; BNP, brain natriuretic peptide; CA, Canadian enhancement; CHF, congestive heart failure; CHS, Cardiovascular Health Study; CM, clinical modification; DRG, Disease Related Group; ED, emergency department; ESC, European Society of Cardiology; HDD, hospital discharge data; HF, heart failure; ICD, International Classification of Disease; IP, inpatient; MRR, medical records review; NHANES, National Health And Nutrition Examination Survey; NYHA, New York Heart Association; NT-pro BNP, B-type natriuretic peptide; OP, outpatient; RCD, routinely collected data; USA, United States of America and WHI, Women’s Health Initiative^. a^ See Table V of for relevant gold standard HF definitions.

Table 2 Characteristics of studies ascertaining prevalent heart failure (ordered by country and number of gold standard events)

| First author,  Year | Country | Year (s) of data collection | Sample size (GS events) | Population | RCD source (s) | RCD type | Diagnostic codes used | Coding positions/ algorithms used (best algorithm in bold) | Gold standard |
| --- | --- | --- | --- | --- | --- | --- | --- | --- | --- |
| Etzioni  2020 [45] | USA | 2013-2015 | 41,432  **(249)** | Hospitalised surgical patients | Local HDD | ICD-9 IP | 398.91, 402.x1, 404.x1, 404.x3, 428.xx | Secondary position present on admission | National Surgical QIP registry data abstracted based on clinical criteria. |
| Rector  2004 [46] | USA | 1999-2000 | 3,633  **(218)** | Adults ≥65 years old | ACD and pharmacy claims data | ICD-9 (CM) IP/OP | 398.91, 402.x1, 404.x1, 404.x3, 414.8, 428.x | **One code in any position over 2 years** (19 other algorithms tested) | Patient self-report survey |
| Van Doorn  1999 [47] | USA | 1989-1991 | 524  **(217)** | Patients ≥ 70 years admitted to a general medical ward | Local HDD | ICD-9  IP | 428.x | Any position | MRR by trained research nurse |
| Kieszak  1999 [48] | USA –  Georgia | 1993 | 1,945  **(145)** | Patients hospitalised for carotid endarterectomy | ACD | ICD-9 (CM) IP | 428.x | Any position in the index hospital admission. | MRR for comorbid HF documented by a physician or under current management |
| Fleming  2011 [49] | USA | 2003-2005 | 2,055  **(103)** | Patients aged ≥66 years with breast or p rostate cancer | ACD | ICD-9 IP/OP | 398.91, 402.x1, 404.x1, 404.x3, 428 | Primary/ 2^ry^ positions for 1 year before cancer diagnosis. At least 2 physician/ OP claims, or 1 hospital claim required. | MRR for HF present at or before cancer diagnosis (ranked severe, moderate, or mild) |
| Borzecki  2004 [50] | USA | 1998-1999 | 1,176  **(82)** | Patients with a coded OP hypertension diagnosis in AD | ACD | ICD-9 (CM) OP | 398.91, 402.x1, 404.x1, 404.x3, 414.8, 428.x | Any diagnostic position at least once within 1 year | MRR for a specific mention of a HF diagnosis |
| Wilchesky  2004 [51] | Canada - Alberta | 1995-1996 | 14,980  **(1,057)** | Adults aged ≥66 years | ACD | ICD-9 IP/OP | 428x, 429.3 | Any position | Primary care physician records review |
| Quan  2008 [52] | Canada - Alberta | 2008 | 4,008  **(333)** | Hospitalised patients aged ≥18 years | Local HDD (ICD-10) + recoded medical records (ICD-9) | ICD-9 (CM), -10 (CA) IP | ICD-9: 428.x  ICD-10: I09.9, I11.0, I13.0, I13.2, I25.5, I42.0, I42.5-I42.9, I43.x, I50.x, P29.0 | Any position | MRR (Charlson ^a^ criteria) - presence of CHF |
| Xu  2020 [53] | Canada - Alberta | 2015 | 2,106  **(296)** | Hospitalised patients (aged ≥18 years) | National HDD and electronic MR database | ICD-10 IP | I09.9, I11.0, I13.0, I13.2, I25.5, I42.0, I42.5, I42.9, I43.x, I50.x, P29.0 | 1: Any position (HDD)  2-8: **Machine learning algorithm**/ keyword searches of discharge summaries or structured EMR data **+**/- **HDD** | MRR using study specific criteria |
| Schultz  2013 [54] | Canada -Ontario | 1. 2004-2005  2. 2007 | 3,441  **(99)** | Patients aged ≥38 years | National HDD and ACD (national ambulatory care data and physician billing claims data) | ICD-8, -9 and -10 IP/ OP | ICD-9 428 or ICD-10 I50.0, I50.1, I50.9 **(narrow)** +/-  ICD-9 425, 514, 518.4 or ICD-10 I42.0, J81 (broad) | Nine algorithms, (1ry position for HDD/ claims, any position for ambulatory care data)  1-3: One code from HDD only/ claims only/any source.  4-6: **One HDD or 1 claims code + 2^nd^ code from any source within 1**/2/3 yrs.  7-9: Two claims /ambulatory care codes over 1/2/3 yrs. | Electronic primary care records review (over a 3-year period) – definite, possible or no HF. |
| Humphries  2000 [55] | Canada - Vancouver | 1994-1995 | 817  **(58)** | Adults undergoing percutaneous coronary interventions | Regional HDD | ICD-9 (CM) IP | 428 | Any position (comorbidity) | MRR for a diagnosis of congestive HF |
| Quan  2002 [56] | Canada - Alberta | 1996-1997 | 1,200  **(128)** | Patients hospitalised under a general physician/ surgeon | Local HDD | ICD-9 (CM) IP | 428.x | ICD-9 codes as a co-morbidity | MRR (Charlson^a^ criteria) presence of CHF |
| So  2006 [57] | Canada - Alberta | 2003 | 193  **(55)** | Patients aged ≥20 years hospitalised  >1 day with acute myocardial infarction | Regional HDD | ICD-9 (CM) or -10 IP | ICD-9 428.x or ICD-10 I09.9, I11.0, I13.0, I13.2, I25.5, I42.2, I42.5-I42.9, I43.x, I50.x, P29.0 | ICD-9 and -10 codes in secondary position (comorbidity) | MRR using study specific criteria |
| Soo  2014 [58] | Scotland | 2003 | 3426  **(546)** | Patients aged >15 years with chronic kidney disease at screening | National HDD | ICD-10 IP | I50.x, I11.0, I13.0, I13.2, I25.5, I42.0, I42.5–I42.9, I43.x | ICD-10 codes as a comorbidity for 5 years prior to screening | MRR for HF present any time before the date of screening. |
| Kaspar  2018 [59] | Germany | 2000-2015 | 1042  **(222)** | Patients admitted to the medical department | Local HDD and electronic MR database | ICD-10 IP | I13.2, I13.0, I11, I50  +/- machine learning algorithm | 1. Any position  2. Any position + machine learning algorithm of unstructured text entries | MRR by cardiologists to identify prevalent HF |
| Luthi  2007 [60] | Switzerland | 2002-2003 | 890  **(52)** | Adults hospitalised from acute care wards | Regional HDD | ICD-10 IP | I09.9, I11,0, I13,0, I13.2, I25.5, I42.0, I42.5-I42.9, I43x, I50x, P29.0 | Any position (comorbidity) | MRR on a single day (study specific criteria) |
| Henderson  2006 [61] | Australia  -Victoria | 1998-1999  2000-2001 | 7,004 **(392)**  7,631 **(153)** | Hospitalised patients | Regional HDD | ICD-10 (AM) IP | I50 | Any position | MRR and recoding by professional coders |
| Powell  2001 [62] | Australia  (NSW) | 1996-1998 | 1765  **(172)** | Patients aged 20-85 years hospitalised for heart disease | Local HDD | ICD-9 (CM) IP | 428 | Secondary position | MRR (study specific criteria) |
| Preen  2004 [63] | Western Australia | 1991-1996 | 2,037  **(100)** | Hospitalised patients | Regional HDD | ICD-9 (CM) IP | Not defined | Any position within 5 years of index admission | MRR |
| Sarfati  2010 [64] | New Zealand | 1996-2003 | 569  **(64)** | Patients aged ≥ 25 years with primary colon carcinoma | National HDD | ICD-9 (CM-A) IP | 428.X | Primary and secondary positions | MRR (study specific criteria) |
| Chong  2011 [65] | Singapore | 2007 | 3,471  **(469)** | Adults aged ≥ 55 years hospitalised for pneumonia | National HDD | ICD-9 (CM) IP | Not defined | Secondary positions for index admission | MRR for physician documented comorbidities during the first 24 hours of admission |

ACD indicates administrative claims data; HDD, hospital discharge data; HF, heart failure; ICD, International Classification of Disease; IP, inpatient; MRR, medical records review; OP, outpatient; QIP, Quality Improvement Programme and RCD, routine healthcare data.

^a^ See Table 5 of for relevant gold standard HF definitions.

Table 3 QUADAS-2 study quality assessment

| **Study details** | | **Risk of bias** | | | | | **Applicability** | | | |
| --- | --- | --- | --- | --- | --- | --- | --- | --- | --- | --- |
| **Author** |  | **Patient selection** | **Index test** | **Reference standard** | **Flow and timing** | **Overall risk of bias** | **Patient selection** | **Index test** | **Reference standard** | **Overall applicability** |
| Allen 2014 | | Low | Low | Unclear | Low | At risk | Low | Low | Low | Low concern |
| Alqaisi 2009 | | Low | Low | Low | Low | Low risk | Low | Low | Low | Low concern |
| Austin 2002 | | Low | Low | Low | Low | Low risk | Low | Low | Low | Low concern |
| Birman-Deych 2005 | | Low | Low | Low | Low | Low risk | Low | Low | Low | Low concern |
| Blackburn 2011 | | Low | Low | Low | Low | Low risk | Low | Low | Low | Low concern |
| Borzecki 2004 | | Low | Low | Low | Low | Low risk | Low | Low | Low | Low concern |
| Bosco-Levy 2019 | | Low | Low | Low | Low | Low risk | Low | Low | Low | Low concern |
| Chong 2011 | | Unclear | Low | Unclear | Low | At risk | Low | Low | Unclear | Some concern |
| Cozzolino 2019 | | Unclear | Low | Low | Low | At risk | Low | Low | Low | Low concern |
| Fisher 1992 | | Low | Low | High | Low | At risk | Low | Low | High | Some concern |
| Fleming 2011 | | Low | Low | Low | Low | Low risk | Low | Low | Low | Low concern |
| Fonseca 2008 | | Low | Low | Low | Low | Low risk | Low | Low | Low | Low concern |
| Frolova 2015 | | Low | Low | Low | Low | Low risk | Low | Low | Low | Low concern |
| Goff 2000 | | Low | Low | Low | Low | Low risk | Low | Low | Low | Low concern |
| Heckbert 2004 | | Unclear | Low | Low | Low | At risk | Low | Low | Low | Low concern |
| Henderson 2006 | | Unclear | Low | High | Low | At risk | Low | Low | High | Some concern |
| Huang 2017 | | Unclear | Low | Low | Low | At risk | Low | Low | Low | Low concern |
| Humphries 2000 | | Unclear | Low | Low | Low | At risk | Low | Low | Low | Low concern |
| Ingelsson 2005 | | Unclear | Low | Low | Unclear | At risk | Low | Low | Low | Low concern |
| Jollis 1993 | | Unclear | Low | Low | Low | At risk | Low | Low | Low | Low concern |
| Juurlink 2006 | | Unclear | Low | High | Low | At risk | Unclear | Low | High | Some concern |
| Khand 2005 | | Low | Low | Low | Unclear | At risk | Low | Low | Low | Low concern |
| Kieszak 1999 | | Low | Low | Low | Low | Low risk | Unclear | Low | Low | Some concern |
| Kumler 2008 | | Low | Low | Low | Low | Low risk | Low | Low | Low | Low concern |
| Lee 2005 | | Unclear | Low | Low | Unclear | At risk | Low | Low | Low | Low concern |
| Li 2011 | | Low | Low | High | Low | At risk | Low | Low | High | Some concern |
| Mahonen 2013 | | Low | Low | Low | Low | Low risk | Low | Low | Low | Low concern |
| Mard 2010 | | Low | Low | Low | Low | Low risk | Low | Low | Low | Low concern |
| MCollough 2002 | | Low | Low | Low | Low | Low risk | Low | Low | Low | Low concern |
| Merry 2009 | | Low | Low | Low | Low | Low risk | Low | Low | Low | Low concern |
| Ono 2020 | | Unclear | Low | Unclear | Unclear | At risk | Low | Low | Unclear | Some concern |
| Powell 2001 | | Low | Low | Low | Low | Low risk | Low | Low | Low | Low concern |
| Preen 2004 | | Low | Low | Low | Unclear | At risk | Low | Low | Low | Low concern |
| Psaty 2016 | | Low | Low | Low | Low | Low risk | Low | Low | Low | Low concern |
| Quan 2008 | | Low | Low | High | Low | At risk | Low | Low | High | Some concern |
| Quan 2002 | | Low | Low | Low | Low | Low risk | Low | Low | Low | Low concern |
| Rector 2004 | | Unclear | Low | High | Low | At risk | Unclear | Low | High | Some concern |
| Roger 2004 | | Low | Low | Low | Low | Low risk | Low | Low | Low | Low concern |
| Sarfati 2010 | | Low | Unclear | Unclear | Low | At risk | Low | Low | Unclear | Some concern |
| Schaufelberger 2020 | | Low | Low | Low | Unclear | At risk | Low | Low | Low | Low concern |
| Schellenbaum 2006 | | Low | Unclear | Low | Low | At risk | Low | Unclear | Low | Some concern |
| Schultz 2013 | | Low | Low | Low | Low | Low risk | Low | Low | Low | Low concern |
| So 2006 | | Low | Low | Low | Unclear | At risk | Low | Low | Low | Low concern |
| Soo 2014 | | Low | Low | Low | Low | Low risk | Low | Low | Low | Low concern |
| Teng 2008 | | Low | Unclear | Unclear | Low | At risk | Low | Low | Low | Low concern |
| Wilchesky 2004 | | Low | Low | Low | High | At risk | Low | Low | Low | Low concern |
| Xu 2020 | | Low | Unclear | Low | Unclear | At risk | Low | Unclear | Low | Some concern |
| Cohen 2020 | | Unclear | Low | Low | Unclear | At risk | Low | Unclear | Low | Some concern |
| Delekta 2018 | | Low | Low | Low | Low | Low risk | Low | Low | Low | Low concern |
| Etzioni 2020 | | Low | Low | Unclear | Low | At risk | Low | Low | Unclear | Some concern |
| Kaspar 2018 | | Low | Low | Low | Low | Low risk | Low | Low | Low | Low concern |
| Luthi 2007 | | Low | Low | Unclear | Low | At risk | Low | Low | Unclear | Some concern |
| Pfister 2013 | | Low | Low | Low | Low | Low risk | Low | Low | Low | Low concern |
| Presley 2018 | | Low | Low | Low | Low | Low risk | Unclear | Low | Low | Some concern |
| Rosamond 2012 | | Unclear | Low | Low | Low | At risk | Low | Low | Low | Low concern |
| Sundboll 2016 | | Low | Low | Low | Unclear | At risk | Low | Low | Low | Low concern |
| Thygesen 2011 | | Low | Low | Low | Low | Low risk | Low | Low | Low | Low concern |
| van Doorn 2001 | | Low | Low | Low | Low | Low risk | Low | Low | Low | Low concern |

Table 4 Sources of routine and gold standard data by country or region

| Data Sources | Number of studies | | | | | | Number (n), percentage (%) of studies using each data source | |
| --- | --- | --- | --- | --- | --- | --- | --- | --- |
|  | **USA**  **n=22** | **Canada**  **n=12** | **Europe**  **n=17** | **Oceania**  **n= 5** | **Asia**  **n=2** | **Total**  **n= 58** | **n** | **%** |
| Routine data source |  |  |  |  |  |  |  |  |
| Hospital discharge data (patient registers, hospital discharge/ morbidity data, ambulatory care data) | **8** | **9 (2) ^a^** | **15 (2)** | **5** | **1** | **38 (4)** | **42** | **72.4** |
| - Local | 5 | 3 | 3 (1) | 1 |  | 12 (1) | 13 | 22.4 |
| - Regional | 3 | 2 | 2 | 3 |  | 10 | 10 | 17.2 |
| - National |  | 4 (2) | 10 | 1 | 1 | 16 (2) | 18 | 31.0 |
| Administrative claims data (physician billing, insurance, hospital or medical services claims) | **7 (4)** | **1 (1)** | **(1)** |  | **1** | **9 (6)** | **15** | **25.9** |
| Pharmacy prescribing, dispensing or drug reimbursement data | **(2)** |  | **(1)** |  |  | **(3)** | **3** | **5.2** |
| Disease or death registry | **3 (1)** |  | **(1)** |  |  | **3 (2)** | **5** | **8.6** |
| Other (laboratory data/ electronic medical records) | **(1)** | **(1)** | **(1)** |  |  | **(3)** | **3** | **5.2** |
| Combined sources | **4** | **2** | **2** |  |  | **8** | **8** | **13.8** |
| Gold standard data source |  |  |  |  |  |  |  |  |
| - Medical records/ electronic medical records | **18** | **9** | **15** | **5** | **2** | **49** | **49** | **84.5** |
| - Specialty database/ registry | **3** | **1** | **1** |  |  | **5** | **5** | **8.6** |
| - Participant self-report | **1** |  |  |  |  | **1** | **1** | **1.7** |
| - Primary care records |  | **2** |  |  |  | **2** | **2** | **3.5** |
| - Prospective history and examination |  |  | **1** |  |  | **1** | **1** | **1.7** |

USA indicates United States of America.  ^a^ Data are shown as number of studies using each data source, with the number in brackets indicating the number of additional studies using multiple data sources including the source in question.

Table 5 Gold standard heart failure ascertainment methods used in the reviewed studies

| Gold standard method | Number (n) and percentage (%) of studies using each criterion | |
| --- | --- | --- |
|  | **n** | % |
| Clinician review of medical/ primary care/ registry records | **49** | **84.5%** |
| - Study specific criteria | 24 | 41.4% |
| - European Society of Cardiology (ESC) criteria ^a^ | 10 ^b^ | 17.2% |
| - Framingham criteria (1971)[66] | 7 ^c^ | 12.1% |
| - Charlson definition (1987)[67] | 2 | 3.5% |
| - Carlson (Boston) criteria (1985)[68] | 2 | 3.5% |
| - CHS adjudication criteria (2004)[69] | 2 | 3.5% |
| - ACC/ AHA criteria [70] | 1 | 1.7% |
| - WHI adjudication criteria[71] | 1 | 1.7% |
| No additional adjudication: | **9** | **15.5%** |
| - Recoding of medical or primary care records by trained coders | 3 | 5.2% |
| - Cardiovascular disease registry diagnosis | 2 | 3.5% |
| - Coded EMR diagnosis | 1 | 1.7% |
| - Ejection fraction only (registry data) | 1 | 1.7% |
| - Patient self-report survey | 1 | 1.7% |
| - Prospective history and examination | 1 | 1.7% |

ACC/AHA indicates, American College of Cardiology/ American Heart Association; CHS, Cardiovascular Health Study; EMR, electronic medical records; and WHI, Women’s Health Initiative. ^a^ Each study used the most up to date ESC guideline at the time of study adjudication.

^b^ Allen et al.[22] also used ACC/AHA left ventricular dysfunction criteria. [72] ^c^ McCollough et al.[21] also used National Health And Nutrition Examination Survey (NHANES) 1 criteria. [73]

Table 6 Guidelines used for gold standard adjudication

| Guideline | Criteria | Scoring system |
| --- | --- | --- |
| European Society of Cardiology (ESC) guidelines (2008)[74] | - Symptoms typical of HF (breathlessness at rest or on exercise, fatigue, tiredness, ankle swelling) and, - Signs typical of HF (tachycardia, tachypnoea, pulmonary rales, pleural effusion, raised JVP, peripheral oedema, hepatomegaly) + - Objective evidence of a structural or functional abnormality of the heart at rest (cardiomegaly, S3, cardiac murmurs, abnormality on the echocardiogram, raised BNP/ NT-pro-BNP) | The presence of clinical signs and symptoms along with objective evidence of structural abnormality of the heart required. Response to treatment also considered. |
| Framingham criteria (1971)[66] | - Major: Orthopnoea/paroxysmal nocturnal dyspnoea (PND), neck vein distension, rales, cardiomegaly, acute pulmonary oedema, S3 gallop, JVP >16cmH_2_O, circulation time ≥ 25s, hepatojugular reflux. - Minor: Ankle oedema, nocturnal cough, dyspnoea on exertion, hepatomegaly, pleural effusion, vital capacity ↓1/3 from maximum, tachycardia (≥120/min), weight loss in response to treatment (≥4.5kg in 5 days) | Definite HF: 2 major or 1 major and 2 minor criteria to present concurrently |
| ACC/AHA guidelines (2014) [70] | - Symptoms: dyspnoea, decreased exercise tolerance, fatigue, worsened end-organ perfusion or other symptoms of volume overload - Physical examination: peripheral oedema, increasing abdominal distention or ascites (in the absence of primary hepatic disease), pulmonary rales/crackles/crepitations, increased JVP and/or hepatojugular reflux, S3 gallop, or clinically significant/ rapid weight gain related to fluid retention (>3–4 lb in 3–4 days) - Investigations: Increased BNP or NT-pro-BNP concentrations,   radiological evidence of pulmonary congestion, non-invasive diagnostic evidence of HF (echocardiography, cardiac MRI, cardiac positron emission tomography scan, and nuclear imaging), invasive diagnostic evidence of HF (right-sided heart catheterization) | At least 1 symptom + at least 2 physical examination findings or 1 examination and 1 laboratory criterion required |
| Women’s Health Initiative (WHI) criteria (2003)[71] | - Signs and symptoms of HF AND - Pulmonary oedema on CXR; OR dilated ventricle or poor LV function on imaging studies; OR physician diagnosis of CHF and receiving medical treatment for CHF | Signs and symptoms plus at least one other criterion required to diagnose CHF. |
| Charlson definition (1987)[42] | - Exertional or PND and - Response symptomatically (or on physical examination) to digitalis, diuretics or afterload reducing agents | Requires both symptoms and response to treatment. Excludes patients on medication with no evidence of clinical improvement. |
| Cardiovascular Health Study (CHS) criteria (2006)[69] | - Physician diagnosis of CHF and, - Symptoms and signs of CHF (shortness of breath, fatigue, orthopnoea, PND, oedema, rales, S3 gallop, displaced apical impulse) and - Supporting clinical evidence (e.g., pulmonary oedema on CXR) or - Medical therapy for CHF (e.g., diuretics, ACE-inhibitors, beta-blockers) | Medical records need to contain a physician diagnosis and symptoms/ signs of HF and supporting clinical evidence or medical therapy to confirm incident HF |
| Carlson (Boston) Criteria (1985)[68] | - History: rest dyspnoea, orthopnoea, PND, dyspnoea on walking on level area, dyspnoea while climbing - Physical examination: Heart rate abnormality, JVP elevation +/- hepatomegaly or oedema, lung crackles, wheezing, S3 - CXR: alveolar or interstitial pulmonary oedema, bilateral pleural effusion CT ratio ≥ 0.50, upper zone flow redistribution | Complex scoring system with a maximum score of 4 for each category. Definite HF 8-12 points, possible HF 5-7 points, and HF unlikely ≤4. |
| NHANES-1 score (1992)[73] | - History: dyspnoea/ difficulty breathing (four levels of activity) - Physical examination: heart rate (91-110/min or ≥111/min) rales/crackles (upper vs. lower lung field), jugular venous distension +/- oedema +/- hepatomegaly - CXR: cephalization of pulmonary vessels, interstitial oedema, +/- pleural fluid, alveolar fluid + pleural fluid | Different scores for each category. CHF defined as a total CHF score ≥3. |

ACE indicates angiotensin converting enzyme; BN, Brain natriuretic peptide; CHF, congestive heart failure; CXR, chest x-ray; LV, left ventricular; MRI, magnetic resonance imaging; NT-proBNP, N-terminal pro B-type natriuretic peptide; PND, paroxysmal nocturnal dyspnoea and S3, 3^rd^ heart sound.

Table 7 ICD-9 coding algorithms used to define heart failure in the studies reviewed

| 428 Heart Failure | Other acute rheumatic HD | Rheumatic HF (congestive) | 402 Hypertensive HD | 404 Hypertensive heart and chronic kidney disease | | | | | | Other specified forms of chronic IHD | Acute cor pulmonale | Chronic pulmonary HD, unspecified | 422 Acute myocarditis | | 425 Cardiomyopathy | | | Cardiomegaly | Functional disturbances following cardiac surgery | Pulmonary congestion and hypostasis | Acute oedema of lung, unspecified | Dyspnoea and respiratory abnormalities | Cardiac complications, NEC | Number of studies (participants with GS HF events) |
| --- | --- | --- | --- | --- | --- | --- | --- | --- | --- | --- | --- | --- | --- | --- | --- | --- | --- | --- | --- | --- | --- | --- | --- | --- |
|  |  |  |  | Hypertensive HD and CKD | Malignant | Benign | Unspecified | With CKD stages I-IV | With CKD stage V or ESRD |  |  |  | Acute myocarditis | Other and unspecified | Cardiomyopathy | Other primary | Secondary, unspecified |  |  |  |  |  |  |  |
| 428.x | **391.8** | **398.91** | **402.x1^a^** | **404.x** | **404.0** | **404.1** | **404.9** | **404.x1 ^a^** | **404.x3 ^a^** | **414.8** | **415.0** | **416.9** | **422.x** | **422.9** | **425.x** | **425.4** | **425.9** | **429.3** | **429.4** | **514** | **518.4** | **786.0** | **997.1** |  |
| X^b,c^ |  |  |  |  |  |  |  |  |  |  |  |  |  |  |  |  |  |  |  |  |  |  |  | 17 (12,373) ((df((1237140) |
| X |  | **X** | **X** |  |  |  |  | **X** | **X** |  |  |  |  |  |  |  |  |  |  |  |  |  |  | 4 (11,584) |
| X |  | **X** | **X** |  |  |  |  |  |  |  |  |  |  |  |  |  |  |  |  |  |  |  |  | 2 (2,048)  (2048) |
| X |  |  | **X** |  |  |  |  | **X** | **X** |  |  |  |  |  |  |  |  |  |  |  |  |  |  | 1 (1,863) |
| X |  | **X** | **X** | **X** | X | X | X | X | X |  | **X** | **X** |  |  |  | **X** |  |  | **X** | **X** | **X** | **X** |  | 1 (1,376) |
| X |  |  |  |  |  |  |  |  |  |  |  |  |  |  |  |  |  | **X** |  |  |  |  |  | 1 (1,057) |
| X | **X** | **X** | **X** |  |  | **X** |  |  |  |  |  |  |  |  | **X** | X | X |  |  | **X** | **X** |  |  | 1 (1,001) |
| X |  |  |  |  |  |  |  |  |  |  |  |  |  |  | **X** | X | X |  |  |  |  |  |  | 1 (795) |
| X |  | **X** | **X** |  |  |  |  |  |  |  |  |  |  |  | **X** | X | X |  |  |  |  |  | **X** | 1 (712) |
| X |  |  | **X** |  |  |  |  | **X** |  |  | **X** | **X** |  |  |  | **X** |  |  |  | **X** | **X** |  |  | 1 (369) |
| X |  | **X** | **X** |  |  |  |  | **X** | **X** |  |  |  |  |  | **X** | X | X |  |  |  |  |  |  | 1 (360) |
| X |  |  | **X** ^d^ |  |  |  |  |  |  | **X** |  |  |  |  |  |  |  |  |  |  |  |  |  | 1 (313) |
| X |  | **X** | **X** |  | **X** | **X** | **X** | **X** | **X** |  |  |  |  |  |  |  |  |  |  |  |  |  |  | 1 (200) |
| X |  | **X** | **X** |  |  |  |  | **X** | **X** |  |  |  |  | **X** | **X** | X | X |  |  |  |  |  |  | 1 (108) |
| X |  |  |  |  |  |  |  |  |  |  |  |  |  |  | **X** | X | X |  |  | **X** | **X** |  |  | 1 (99) |
| X |  | **X** | **X** |  |  |  |  | **X** | **X** | **X** |  |  |  |  |  |  |  |  |  |  |  |  |  | 1 (82) |

X indicates all codes within that group; CKD, chronic kidney disease; HD, heart disease; HF, heart failure; IHD, ischaemic heart disease; ESRD, end stage renal disease and NEC, not elsewhere classified. ^a^ Code indicates additional congestion or heart failure. ^b^ One study used 428.2x (systolic HF) alone or with 428.4x (combined systolic and diastolic HF) in 2 algorithms.[16] ^c^ One study used 428.3x (diastolic HF) in those with at least one 428 code in the past.[11] ^d^ Code 402.9B used. [33] Three studies did not define the ICD-9 codes used.[37, 63, 65]

Table 8 ICD-10 coding algorithms used to define heart failure in the studies reviewed

| I50 Heart failure | Other acute rheumatic heart disease | Rheumatic chorea with heart involvement | Rheumatic heart disease, unspecified | I11 Hypertensive heart disease | I13 Hypertensive heart and renal disease | | I25.5 Ischaemic cardiomyopathy | I42 Cardiomyopathy | | | | | | | I43 Cardiomyopathy in diseases classified elsewhere | J81 Pulmonary oedema | P29.0 Neonatal cardiac failure | Number of studies (participants with GS HF) |
| --- | --- | --- | --- | --- | --- | --- | --- | --- | --- | --- | --- | --- | --- | --- | --- | --- | --- | --- |
|  |  |  |  |  | With CKD stages I-IV | With CKD stage V or ESRD |  | Cardiomyopathy | Dilated | Other restrictive | Alcoholic | Due to drug and external agent | Other | Unspecified |  |  |  |  |
| I50.x | **I01.8** | **I02.0** | **I09.9** | **I11.0** ^a^ | **I13.0** ^a^ | **I13.2** ^a^ | **I25.5** | **I42.x** | **I42.0** | **I42.5** | **I42.6** | **I42.7** | **I42.8** | **I42.9** | **I43x** | **J81** | **P29.0** | **N** |
| X^b^ |  |  |  |  |  |  |  |  |  |  |  |  |  |  |  |  |  | 9 (6,669) |
| X |  |  |  | **X** | **X** | **X** |  |  |  |  |  |  |  |  |  |  |  | 4 (5,989) |
| X | **X** | **X** |  | **X** | **X** | **X** |  | **X** | X | X | X | X | X | X |  | **X** |  | 1 (1001) |
| X |  |  |  | **X** | **X** | **X** |  |  | **X** |  | **X** | **X** | **X** | **X** |  |  |  | 2 (737) |
| X |  |  | **X** | **X** | **X** | **X** | **X** |  | **X** | **X** | **X** | **X** | **X** | **X** | **X** |  | **X** | 4 (736) |
| X |  |  |  | **X** | **X** | **X** | **X** |  | **X** | **X** | **X** | **X** | **X** | **X** | **X** |  |  | 1 (546) |
| X |  |  |  | **X** |  |  |  |  |  |  |  |  |  |  |  |  |  | 1 (321) |
| X^c^ |  |  |  | **X** |  |  | **X** |  |  |  |  |  |  | **X** |  |  |  | 1 (216) |
| X |  |  |  |  |  |  |  |  | **X** |  |  |  |  |  |  | **X** |  | 1 (99) |

^a^ Code indicates additional congestion or heart failure. X indicates all codes within that group; CKD, chronic kidney disease; ESRD, end stage renal disease and IHD, ischaemic heart disease.

^b^ A narrow definition of I50.3x used in those with at least one I50 code in the past.[11]

^c^ I50.1 and I50.2 specified as the HF codes used.[36]

One study did not define the ICD-10 codes used.[27]

Table 9 List of ICD codes used across the studies and their definitions

| **ICD code type** | **Code** | **Definition** |
| --- | --- | --- |
| **ICD-10** | I01.8 | Other acute rheumatic heart disease |
|  | I02.0 | Rheumatic chorea with heart involvement |
|  | I09.0 | Rheumatic myocarditis |
|  | I09.9 | Rheumatic heart disease, unspecified. |
|  | I11 | Hypertensive heart disease |
|  | I11.0 | Hypertensive heart disease with (congestive) heart failure |
|  | I11.1 | Listed as dilated cardiomyopathy in Khand et al. but not in ICD-10[36] |
|  | I13 | Hypertensive heart and renal disease |
|  | I13.0 | Hypertensive heart and renal disease with (congestive) heart failure |
|  | I13.2 | Hypertensive heart and renal disease with (congestive) heart failure and renal failure |
|  | I25.5 | Ischemic cardiomyopathy |
|  | I42 | Cardiomyopathy |
|  | I42.0 | Dilated cardiomyopathy |
|  | I42.1 | Obstructive hypertrophic cardiomyopathy |
|  | I42.2 | Other hypertrophic cardiomyopathy |
|  | I42.3 | Endomyocardial (eosinophilic) disease |
|  | I42.4 | Endocardial fibroelastosis |
|  | I42.5 | Other restrictive cardiomyopathy |
|  | I42.6 | Alcoholic cardiomyopathy |
|  | I42.7 | Cardiomyopathy due to drug and external agent |
|  | I42.8 | Other cardiomyopathies |
|  | I42.9 | Cardiomyopathy, unspecified |
|  | I43 | Cardiomyopathy in diseases classified elsewhere |
|  | I43.x | Cardiomyopathy in diseases classified elsewhere |
|  | I43.0 | Cardiomyopathy in infectious and parasitic diseases classified elsewhere |
|  | I43.1 | Cardiomyopathy in metabolic diseases |
|  | I43.2 | Cardiomyopathy in nutritional diseases |
|  | I43.8 | Cardiomyopathy in other diseases classified elsewhere |
|  | I50 | Heart failure |
|  | I50.x | Heart failure |
|  | I50.0 | Congestive heart failure |
|  | I50.1 | Left ventricular failure |
|  | I50.2***** | Systolic congestive heart failure |
|  | I50.3***** | Diastolic (congestive) heart failure |
|  | I50.4***** | Combined systolic (congestive) and diastolic (congestive) heart failure |
|  | I50.9 | Heart failure, unspecified |
|  | J81 | Pulmonary oedema |
|  | P29.0 | Neonatal cardiac failure​ |
|  | V42.1 | Heart replaced by transplant |
| **ICD-9** | 398.91 | Rheumatic heart failure (congestive) |
|  | 402.0 | Malignant hypertensive heart disease |
|  | 402.x1 | Benign, malignant or unspecified hypertensive heart disease with heart failure |
|  | 402.x3 | Listed in Allen et al. but not in ICD-9[22] |
|  | 402.01 | Malignant hypertensive heart disease with heart failure |
|  | 402.11 | Benign hypertensive heart disease with heart failure |
|  | 402.91 | Unspecified hypertensive heart disease with heart failure |
|  | 404.0 | Malignant hypertensive heart and renal disease |
|  | 404.x | Malignant hypertensive heart and renal disease |
|  | 404.x3 | Hypertensive heart and chronic kidney disease, benign, malignant or unspecified, with heart failure and with chronic kidney disease stage V or end stage renal disease |
|  | 404.01 | Hypertensive heart and chronic kidney disease, malignant, with heart failure and with chronic kidney disease stage I through stage IV, or unspecified |
|  | 404.03 | Hypertensive heart and chronic kidney disease, malignant, with heart failure and with chronic kidney disease stage V or end stage renal disease |
|  | 404.10 | Hypertensive heart and chronic kidney disease, benign, without heart failure and with chronic kidney disease stage I through stage IV, or unspecified |
|  | 404.11 | Hypertensive heart and chronic kidney disease, benign, with heart failure and with chronic kidney disease stage I through stage IV, or unspecified |
|  | 404.13 | Hypertensive heart and chronic kidney disease, benign, with heart failure and chronic kidney disease stage V or end stage renal disease |
|  | 404.90 | Hypertensive heart and chronic kidney disease, unspecified, without heart failure and with chronic kidney disease stage I through stage IV, or unspecified |
|  | 404.91 | Hypertensive heart and chronic kidney disease, unspecified, with heart failure and with chronic kidney disease stage I through stage IV, or unspecified |
|  | 404.93 | Hypertensive heart and chronic kidney disease, unspecified, with heart failure and chronic kidney disease stage V or end stage renal disease |
|  | 414.8 | Other specified forms of chronic ischemic heart disease |
|  | 415.0 | Acute cor pulmonale |
|  | 416.0 | Primary pulmonary hypertension |
|  | 416.9 | Chronic pulmonary heart disease, unspecified |
|  | 422.0 | Acute myocarditis in diseases classified elsewhere |
|  | 422.9 | Acute myocarditis, unspecified |
|  | 425 | Cardiomyopathy |
|  | 425.x | Cardiomyopathy |
|  | 425.00 | Endomyocardial fibrosis |
|  | 425.40 | Other primary cardiomyopathies |
|  | 425.90 | Secondary cardiomyopathy, unspecified |
|  | 428 | Heart failure |
|  | 428.x | Heart failure |
|  | 428.00 | Congestive heart failure |
|  | 428.10 | Left heart failure, acute pulmonary oedema (with heart disease or failure) |
|  | 428.20 | Systolic heart failure |
|  | 428.30 | Diastolic heart failure |
|  | 428.40 | Combined systolic and diastolic heart failure |
|  | 428.90 | Heart failure, unspecified |
|  | 429.3 | Cardiomegaly |
|  | 429.4 | Functional disturbances following cardiac surgery |
|  | 514 | Pulmonary congestion and hypostasis |
|  | 518.4 | Acute oedema of lung, unspecified |
|  | 786.0 | Dyspnoea and respiratory abnormalities |
|  | 997.1 | Cardiac complications, not elsewhere classified |
| **ICD-8** | 427.0 | Congestive heart failure |
|  | 427.1 | Left ventricular failure |
|  | 428 | Other myocardial insufficiency |
|  | 428.99 | Definition unavailable. Used by Ingelsson et al. and Mahonen et al.[32, 33] |

ICD indicates International Classification of Diseases and CM, Clinical Modification.

* ICD-10 CM codes.

Table 10 Summary diagnostic accuracy statistics for coding algorithms ascertaining acute heart failure according to subgroup

| Subgroup/ study characteristic | Algorithms ^a^  (N) | Sensitivity  (95 % CI) | I^2^ for sensitivity (95% CI) | Specificity (95% CI) | I^2^ for specificity (95% CI) |
| --- | --- | --- | --- | --- | --- |
| RCD source |  |  |  |  |  |
| Hospital discharge data | 10 | 69.5% (55.8-80.4) | 99.3 (99.1-99.4) | 97.1% (92.4-98.9) | 99.7 (99.7-99.8) |
| Administrative claims data | 4 | 59.0% (34.6-79.7) | 99.5 (99.4-99.7) | 93.2% (89.2-95.7) | 98.9 (98.4-99.4) |
| RCD algorithm |  |  |  |  |  |
| I50x/428x | 5 | 67.1% (39.5-86.4) | 99.8 (99.7-99.8) | 98.9% (96.1-99.7) | 99.8 (99.8-99.9) |
| Complex (1 source) | 7 | 70.7% (56.7-81.5) | 99.5 (99.4-99.6) | 92.9% (87.8-96.0) | 99.5 (99.3-99.6) |
| GS data source |  |  |  |  |  |
| Medical records | 12 | 72.6% (61.2-81.7) | 99.3 (99.1-99.4) | 95.1% (88.5-98.0) | 99.6 (99.6-99.7) |
| Registry data | 4 | 41.2% (30.3-53.0) | 99.3 (99.0-99.5) | 97.6% (83.7-99.7) | 99.8 (99.8-99.9) |
| GS adjudication  criteria | | | | | |
| Study specific | 7 | 64.5% (51.5-75.8) | 99.3 (99.2-99.5) | 94.6% (85.1-98.2) | 99.6 (99.5-99.7) |
| Guideline criteria | 5 | 63.4% (34.1-85.3) | 99.2 (99.0-99.5) | 95.1% (82.5-98.8) | 99.8 (99.7-99.8) |
| No adjudication | 5 | 62.4% (38.9-81.2) | 99.6 (99.5-99.7) | 98.2% (90.2-99.7) | 99.8 (99.8-99.9) |
| Country/ region |  |  |  |  |  |
| USA | 9 | 60.3% (44.8-74.1) | 99.4 (99.3-99.5) | 94.3% (89.0-97.1) | 99.6 (99.5-99.7) |
| Europe | 4^b^ | 61.3% (25.5-88.1) | 98.8 (98.2-99.3) | 99.1% (95.9-99.9) | 99.5 (99.3-99.6) |
| Year of publication |  |  |  |  |  |
| Before 2010 | 10 | 66.2% (52.2-77.8) | 99.5 (99.4-99.6) | 97.2% (92.3-99.0) | 99.7 (99.6-99.7) |
| 2010-2020 | 7 | 59.6% (37.9-78.2) | 98.9 (98.6-99.2) | 94.1% (81.0-98.3) | 99.7 (99.7-99.8) |
| Study total |  |  |  |  |  |
| <1500 | 4 | 75.3% (41.4-93.0) | 99.3 (99.1-99.6) | 76.1% (63.2-85.4) | 98.7 (98.1-99.3) |
| ≥1500 | 13 | 59.8% (48.2-70.5) | 99.3 (99.3-99.5) | 97.9% (95.4-99.1) | 99.7 (99.6-99.7) |

CI indicates confidence intervals; GS, gold standard; ICD, International Classification of Disease; I^2^, I^2^ statistic describing the percentage of variation across studies that is due to heterogeneity rather than chance N, number of studies (the same study can be included more than once if assessing algorithms fitting in to different subgroups); RCD, routinely collected healthcare data; and USA, United States of America*.*

^a^ Limited analysis as a minimum of 4 studies are needed for subgroup analysis.

^b^ One study was removed as the unusually low specificity was an outlier that prevented the bivariate model from fitting.[37]

##
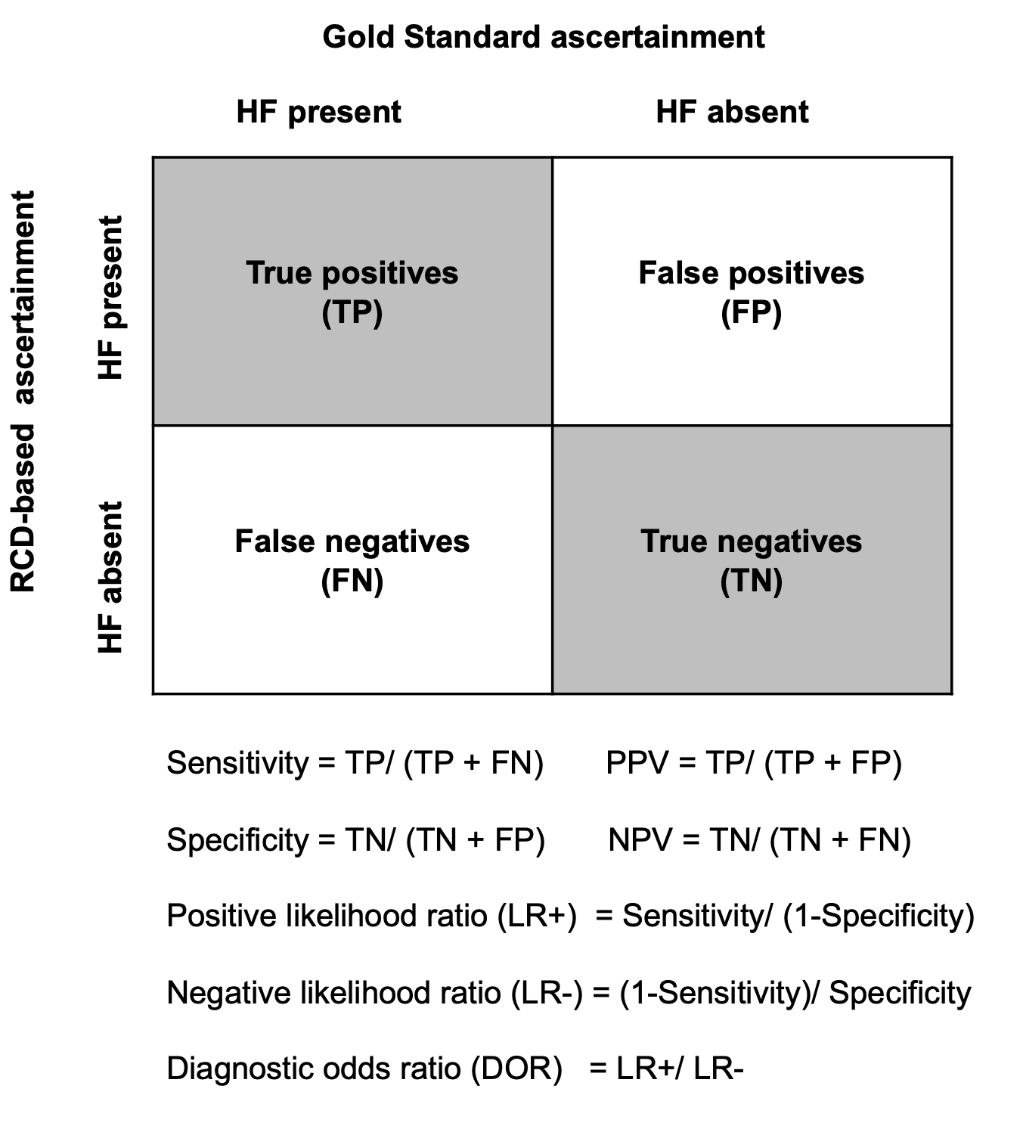
Supplemental Figures

Supplemental Fig. 1 Calculation of performance statistics

Two by two table showing the method of calculating the main performance statistics for each study. HF indicates HF indicates heart failure; NPV, negative predictive values; PPV, positive predictive value and RCD, routinely collected healthcare data.

Supplemental Fig. 2 Funnel plot for the meta-analysis of studies ascertaining acute and prevalent HF using effective sample size weighted regression tests of funnel plot asymmetry


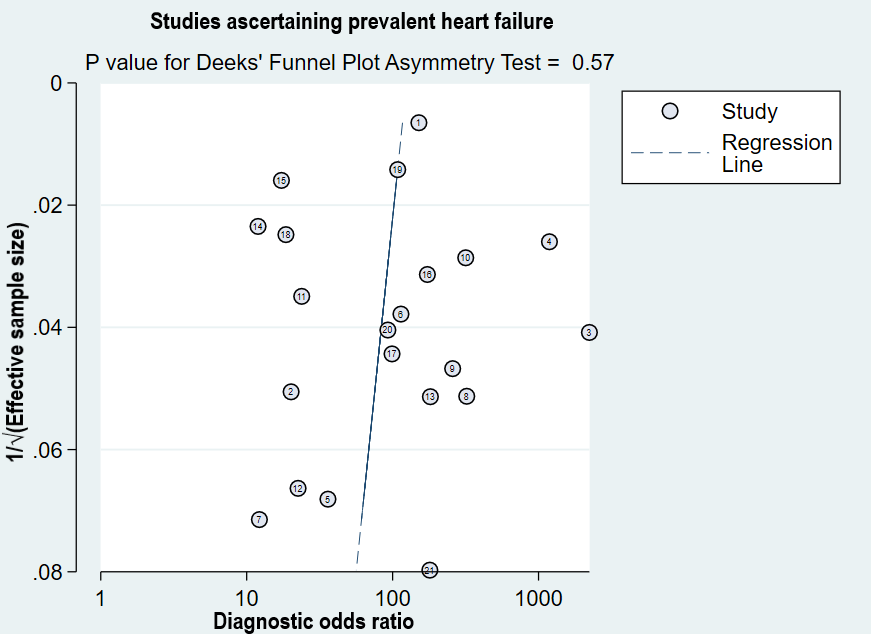

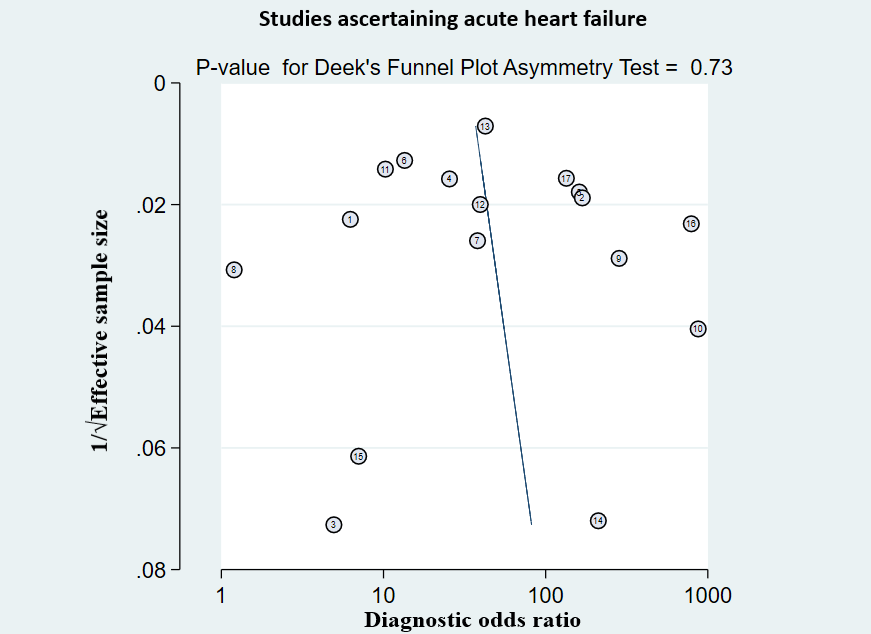


Funnel plots for studies ascertaining acute and prevalent heart failure. Grey circles indicate individual studies, while the blue dashes indicate the regression line. The P-value for the slope co-efficient is estimated using Deek’s funnel plot asymmetry test.[6] 1/√effective sample size is the inverse of the square root of the effective sample size.

Supplemental Fig. 3 SROC plot for the diagnostic accuracy of coding algorithms in studies with > 200 gold standard (GS) heart failure (HF) events

a b

a. Acute HF algorithms and b. Prevalent HF algorithms with >200 GS HF events. HSROC indicates hierarchical summary receiver operating characteristics curve, grey circle, the sensitivity and (1-specificity) of an individual study with the size of the circle proportionate to study size; summary point, summary sensitivity and specificity; 95% confidence region, 95% confidence region for the summary point, and the 95% prediction region, the area in which we can say with 95% certainty the true sensitivity and specificity of a future study will be contained.

Supplemental Fig. 4 SROC plots for the diagnostics accuracy of RCD algorithms ascertaining acute heart failure according to coding position

a
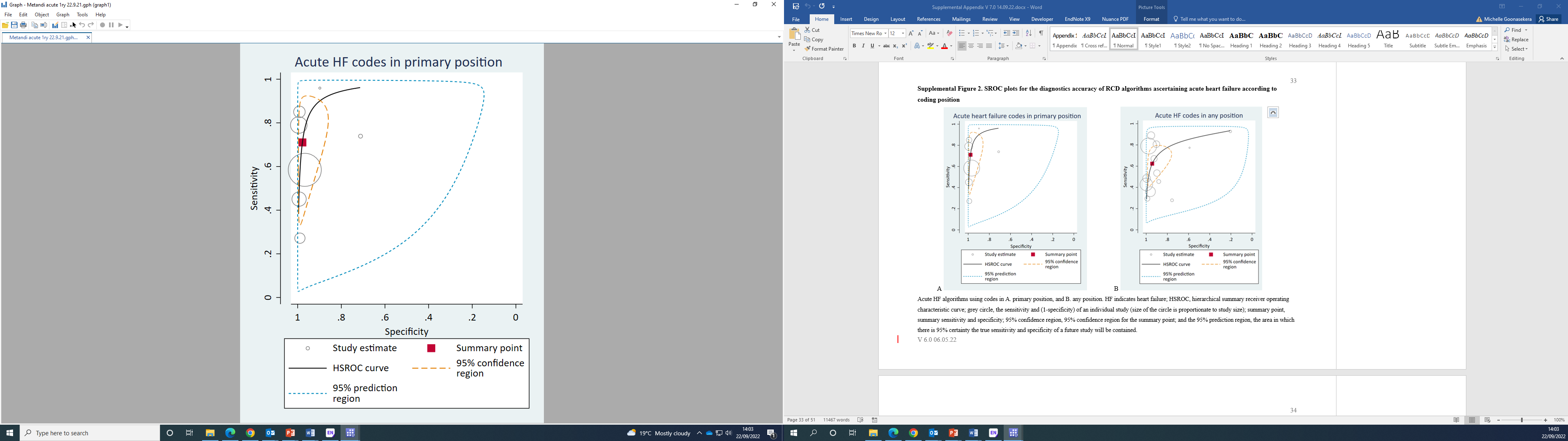
 b
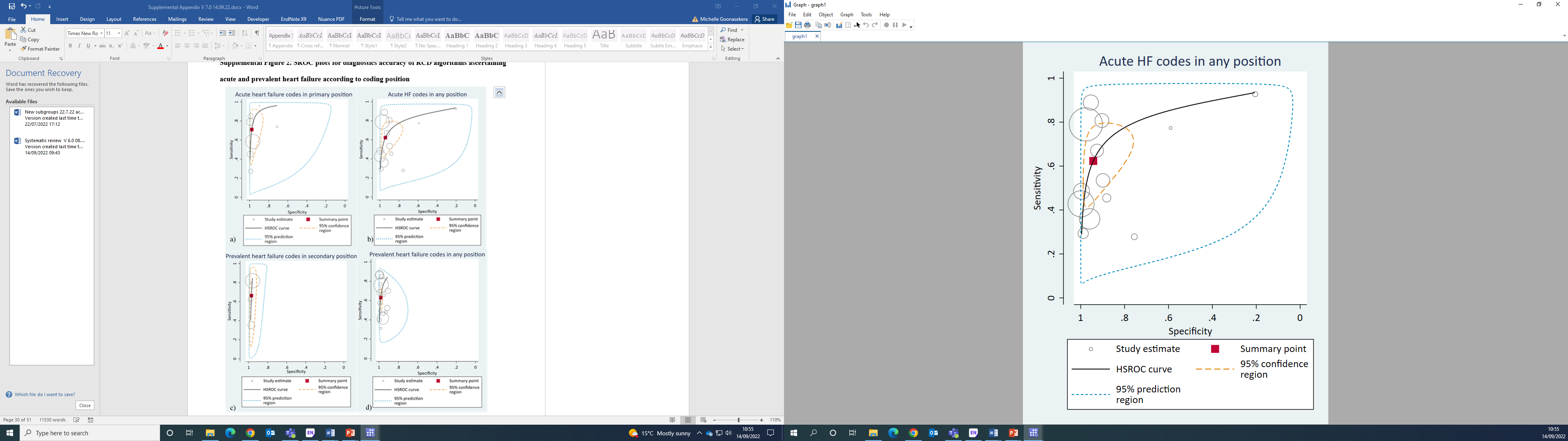


a. Codes in primary position, and b. codes in any position. HF indicates heart failure; HSROC, hierarchical summary receiver operating characteristic curve; grey circle, the sensitivity and (1-specificity) of an individual study (size of the circle is proportionate to study size); summary point, summary sensitivity and specificity; 95% confidence region, 95% confidence region for the summary point; and the 95% prediction region, the area in which there is 95% certainty the true sensitivity and specificity of a future study will be contained.

Supplemental Fig. 5 SROC plots for the diagnostics accuracy of RCD algorithms ascertaining prevalent heart failure according to coding position

a
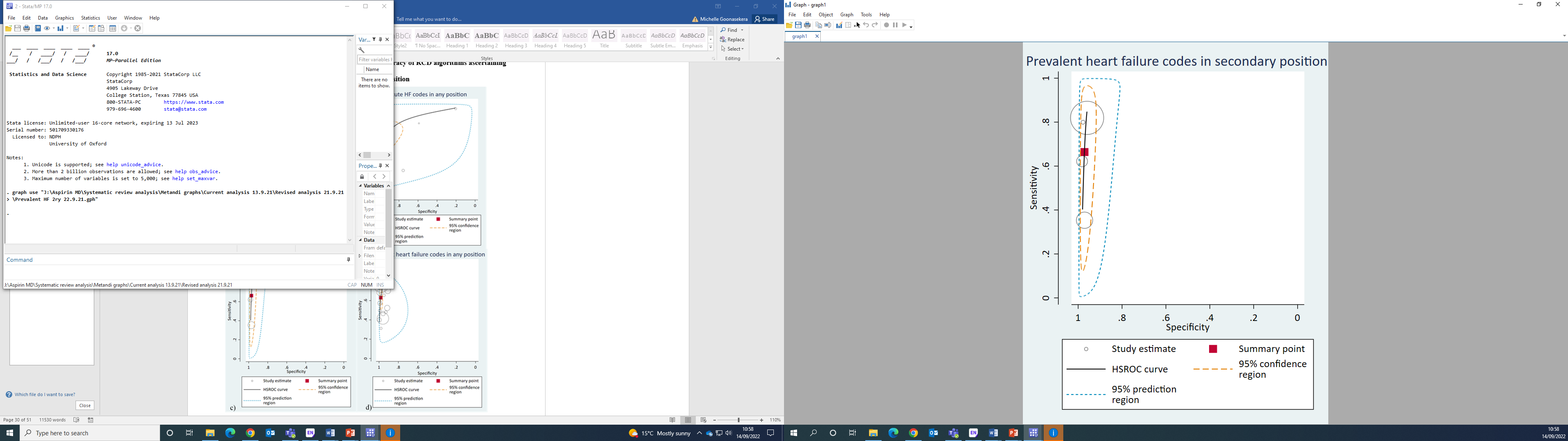
 b
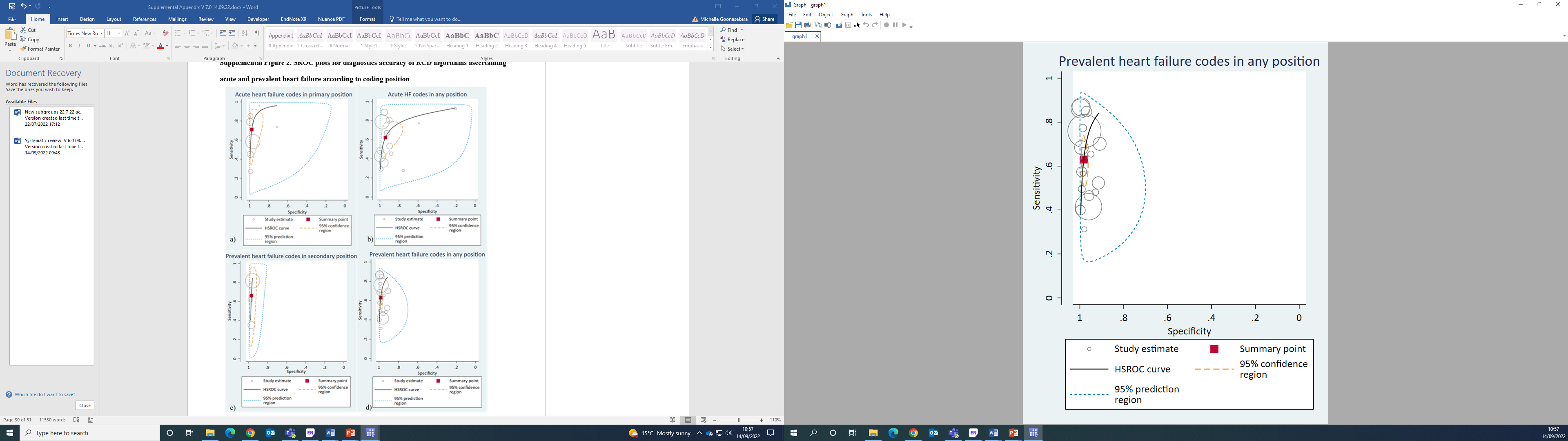


Algorithms using codes in a. secondary positions, and b. any position. HF indicates heart failure; HSROC, hierarchical summary receiver operating characteristic curve; grey circle, the sensitivity and (1-specificity) of an individual study (size of the circle is proportionate to study size); summary point, summary sensitivity and specificity; 95% confidence region, 95% confidence region for the summary point; and the 95% prediction region, the area in which there is 95% certainty the true sensitivity and specificity of a future study will be contained.

## References

1. Seed P. DIAGT: Stata module to report summary statistics for diagnostic tests compared to true disease status. Statistical Software Components. 2010.

2. Reitsma JB, Glas AS, Rutjes AWS, Scholten RJPM, Bossuyt PM, Zwinderman AH. Bivariate analysis of sensitivity and specificity produces informative summary measures in diagnostic reviews. J Clin Epidemiol. 2005;58(10):982-90. doi:10.1016/j.jclinepi.2005.02.022

3. Rutter CM, Gatsonis CA. A hierarchical regression approach to meta-analysis of diagnostic test accuracy evaluations. Stat Med. 2001;20(19):2865-84. doi:10.1002/sim.942

4. Macaskill P GC, Deeks JJ, Harbord RM, Takwoingi Y. Chapter 10: Analysing and Presenting Results. In: Deeks JJ, Bossuyt PM, Gatsonis C (editors), Cochrane Handbook for Systematic Reviews of Diagnostic Test Accuracy Version 1.0.: The Cochrane Collaboration; 2010. Available from: h<ttp://srdta.cochrane.org/.>

5. Dwamena B. MIDAS: Stata module for meta-analytical integration of diagnostic test accuracy studies. Statistical Software Components. 2007.

6. Deeks JJ, Macaskill P, Irwig L. The performance of tests of publication bias and other sample size effects in systematic reviews of diagnostic test accuracy was assessed. Journal of Clinical Epidemiology. 2005;58(9):882-93. doi:h
<ttps://doi.org/10.1016/j.jclinepi.2005.01.016>

7. Birman-Deych E, Waterman AD, Yan Y, Nilasena DS, Radford MJ, Gage BF. Accuracy of ICD-9-CM codes for identifying cardiovascular and stroke risk factors. Med Care. 2005;43(5):480-5. doi:10.1097/01.mlr.0000160417.39497.a9

8. Psaty BM, Delaney JA, Arnold AM, et al. Study of Cardiovascular Health Outcomes in the Era of Claims Data. Circulation. 2016;133(2):156-64. doi:h
<ttp://dx.doi.org/10.1161/CIRCULATIONAHA.115.018610>

9. Jollis JG, Ancukiewicz M, DeLong ER, Pryor DB, Muhlbaier LH, Mark DB. Discordance of databases designed for claims payment versus clinical information systems. Implications for outcomes research. Ann Intern Med. 1993;119(8):844-50. doi:10.7326/0003-4819-119-8-199310150-00011

10. Goff DC, Jr, Pandey DK, Chan FA, Ortiz C, Nichaman MZ. Congestive heart failure in the United States: Is there more than meets the I(CD Code)? The Corpus Christi Heart Project. Arch Intern Med. 2000;160(2):197-202. doi:10.1001/archinte.160.2.197

11. Cohen SS, Roger VL, Weston SA, et al. Evaluation of claims-based computable phenotypes to identify heart failure patients with preserved ejection fraction. Pharmacol Res Perspect. 2020;8(6). doi:10.1002/prp2.676

12. Heckbert SR, Kooperberg C, Safford MM, et al. Comparison of self-report, hospital discharge codes, and adjudication of cardiovascular events in the Women’s Health Initiative. Am J Epidemiol. 2004;160(12):1152-8. doi:10.1093/aje/kwh314

13. Fisher ES, Whaley FS, Krushat WM, et al. The accuracy of Medicare's hospital claims data: progress has been made, but problems remain. Am J Public Health. 1992;82(2):243-8. doi:10.2105/ajph.82.2.243

14. Schellenbaum GD, Heckbert SR, Smith NL, et al. Congestive heart failure incidence and prognosis: case identification using central adjudication versus hospital discharge diagnoses. Ann Epidemiol. 2006;16(2):115-22. doi:10.1016/j.annepidem.2005.02.012

15. Roger VL, Weston SA, Redfield MM, et al. Trends in Heart Failure Incidence and Survival in a Community-Based Population. Jama. 2004;292(3):344-50. doi:10.1001/jama.292.3.344

16. Li Q, Glynn RJ, Dreyer NA, Liu J, Mogun H, Setoguchi S. Validity of claims-based definitions of left ventricular systolic dysfunction in Medicare patients. Pharmacoepidemiol Drug Saf. 2011;20(7):700-8. doi:10.1002/pds.2146

17. Rosamond WD, Chang PP, Baggett C, et al. Classification of Heart Failure in the Atherosclerosis Risk in Communities (ARIC) Study. Circ Heart Fail. 2012;5(2):152-9. doi:10.1161/CIRCHEARTFAILURE.111.963199

18. Huang H, Turner M, Raju S, et al. Identification of acute decompensated heart failure hospitalisations using administrative data. Am J Cardiol. 2017;119(11):1791-6. doi:10.1016/j.amjcard.2017.03.007

19. Presley CA, Min JY, Chipman J, et al. Validation of an algorithm to identify heart failure hospitalisations in patients with diabetes within the veterans health administration. BMJ Open. 2018;8(3):e020455. doi:10.1136/bmjopen-2017-020455

20. Alqaisi F, Williams LK, Peterson EL, Lanfear DE. Comparing methods for identifying patients with heart failure using electronic data sources. BMC Health Serv Res. 2009;9:237. doi:10.1186/1472-6963-9-237

21. McCullough PA, Philbin EF, Spertus JA, et al. Confirmation of a heart failure epidemic: findings from the Resource Utilization Among Congestive Heart Failure (REACH) study. J Am Coll Cardiol. 2002;39(1):60-9. doi:10.1016/S0735-1097(01)01700-4

22. Allen LA, Yood MU, Wagner EH, et al. Performance of claims-based algorithms for identifying heart failure and cardiomyopathy among patients diagnosed with breast cancer. Med Care. 2014;52(5):e30-8. doi:10.1097/MLR.0b013e31825a8c22

23. Austin PC, Daly PA, Tu JV. A multicenter study of the coding accuracy of hospital discharge administrative data for patients admitted to cardiac care units in Ontario. Am Heart J. 2002;144(2):290-6. doi:10.1067/mhj.2002.123839

24. Juurlink D PC, Croxford R, Chong A, Austin P, Tu J, Laupacis A. . Canadian Institute for Health Information Discharge Abstract Database: A Validation Study. Toronto:: Institute for Clinical Evaluative Sciences 2006.

25. Lee DS, Donovan L, Austin PC, et al. Comparison of coding of heart failure and comorbidities in administrative and clinical data for use in outcomes research. Med Care. 2005;43(2):182-8. doi:10.1097/00005650-200502000-00012

26. Frolova N, Bakal JA, McAlister FA, et al. Assessing the use of international classification of diseases-10th revision codes from the emergency department for the identification of acute heart failure. JACC: Heart Fail. 2015;3(5):386-91. doi:10.1016/j.jchf.2014.11.010

27. Blackburn DF, Shnell G, Lamb DA, Tsuyuki RT, Stang MR, Wilson TW. Coding of heart failure diagnoses in Saskatchewan: a validation study of hospital discharge abstracts. J Popul Ther Clin Pharmacol. 2011;18(3):e407-15.

28. Schaufelberger M, Ekestubbe S, Hultgren S, et al. Validity of heart failure diagnoses made in 2000–2012 in western Sweden. ESC Heart Fail. 2020;7(1):37-46. doi:10.1002/ehf2.12519

29. Mard S, Nielsen FE. Positive predictive value and impact of misdiagnosis of a heart failure diagnosis in administrative registers among patients admitted to a University Hospital cardiac care unit. Clin Epidemiol. 2010;2:235-9. doi:10.2147/clep.S12457

30. Delekta J, Hansen SM, AlZuhairi KS, Bork CS, Joensen AM. The validity of the diagnosis of heart failure (I50.0-I50.9) in the Danish National Patient Register. Dan Med J. 2018;65(4).

31. Pfister R, Michels G, Wilfred J, Luben R, Wareham NJ, Khaw K-T. Does ICD-10 hospital discharge code I50 identify people with heart failure? A validation study within the EPIC-Norfolk study. Int J Cardiol. 2013;168(4):4413-4. doi:10.1016/j.ijcard.2013.05.031

32. Ingelsson E, Ärnlöv J, Sundström J, Lind L. The validity of a diagnosis of heart failure in a hospital discharge register. Eur J Heart Fail. 2005;7(5):787-91. doi:10.1016/j.ejheart.2004.12.007

33. Mahonen M, Jula A, Harald K, et al. The validity of heart failure diagnoses obtained from administrative registers. Eur J Prev Cardiol. 2013;20(2):254-9. doi:10.1177/2047487312438979

34. Kümler T, Gislason GH, Kirk V, et al. Accuracy of a heart failure diagnosis in administrative registers. Eur J Heart Fail. 2008;10(7):658-60. doi:10.1016/j.ejheart.2008.05.006

35. Bosco-Levy P, Duret S, Picard F, et al. Diagnostic accuracy of the International Classification of Diseases, Tenth Revision, codes of heart failure in an administrative database. Pharmacoepidemiol Drug Saf. 2019;28(2):194-200. doi:10.1002/pds.4690

36. Khand AU, Shaw M, Gemmel I, Cleland JGF. Do discharge codes underestimate hospitalisation due to heart failure? Validation study of hospital discharge coding for heart failure. Eur J Heart Fail. 2005;7(5):792-7. doi:10.1016/j.ejheart.2005.04.001

37. Fonseca C, Sarmento PM, Marques F, Ceia F. Validity of a discharge diagnosis of heart failure: implications of misdiagnosing. Congest Heart Fail. 2008;14(4):187-91. doi:10.1111/j.1751-7133.2008.07752.x

38. Merry AH, Boer JM, Schouten LJ, et al. Validity of coronary heart diseases and heart failure based on hospital discharge and mortality data in the Netherlands using the cardiovascular registry Maastricht cohort study. Eur J Epidemiol. 2009;24(5):237-47. doi:10.1007/s10654-009-9335-x

39. Cozzolino F, Montedori A, Abraha I, et al. A diagnostic accuracy study validating cardiovascular ICD-9-CM codes in healthcare administrative databases. The Umbria data-value project. PLoS ONE. 2019;14(7). doi:<http://dx.doi.org/10.1371/journal.pone.0218919>

40. Sundbøll J, Adelborg K, Munch T, et al. Positive predictive value of cardiovascular diagnoses in the Danish National Patient Registry: a validation study. BMJ Open. 2016;6(11):e012832. doi:10.1136/bmjopen-2016-012832

41. Thygesen SK, Christiansen CF, Christensen S, Lash TL, Sørensen HT. The predictive value of ICD-10 diagnostic coding used to assess Charlson comorbidity index conditions in the population-based Danish National Registry of Patients. BMC Med Res Methodol. 2011;11:83. doi:10.1186/1471-2288-11-83

42. Charlson ME, Pompei P, Ales KL, MacKenzie CR. A new method of classifying prognostic comorbidity in longitudinal studies: Development and validation. J Chronic Dis. 1987;40(5):373-83. doi:10.1016/0021-9681(87)90171-8

43. Teng T-HK, Finn J, Hung J, Geelhoed E, Hobbs M. A validation study: how effective is the Hospital Morbidity Data as a surveillance tool for heart failure in Western Australia? Aust N Z J Public Health. 2008;32(5):405-7. doi:10.1111/j.1753-6405.2008.00269.x

44. Ono Y, Taneda Y, Takeshima T, Iwasaki K, Yasui A. Validity of Claims Diagnosis Codes for Cardiovascular Diseases in Diabetes Patients in Japanese Administrative Database. Clin Epidemiol. 2020;12:367-75. doi:10.2147/CLEP.S245555

45. Etzioni DA, Lessow C, Bordeianou LG, et al. Concordance between registry and administrative data in the determination of comorbidity: A multi-institutional study. Ann Surg. 2020;272(6):1006-11. doi:10.1097/SLA.0000000000003247

46. Rector TS, Wickstrom SL, Shah M, et al. Specificity and sensitivity of claims-based algorithms for identifying members of Medicare+Choice health plans that have chronic medical conditions. Health Serv Res. 2004;39(6 Pt 1):1839-57. doi:10.1111/j.1475-6773.2004.00321.x

47. van Doorn C, Bogardus ST, Williams CS, Concato J, Towle VR, Inouye SK. Risk adjustment for older hospitalized persons: A comparison of two methods of data collection for the Charlson index. J Clin Epidemiol. 2001;54(7):694-701. doi:10.1016/S0895-4356(00)00367-X

48. Kieszak SM, Flanders WD, Kosinski AS, Shipp CC, Karp H. A comparison of the Charlson Comorbidity Index derived from medical record data and administrative billing data. J Clin Epidemiol. 1999;52(2):137-42. doi:10.1016/S0895-4356(98)00154-1

49. Fleming ST, Sabatino SA, Kimmick G, et al. Developing a claim-based version of the ACE-27 comorbidity index: a comparison with medical record review. Med Care. 2011;49(8):752-60. doi:10.1097/MLR.0b013e318215d7dd

50. Borzecki AM, Wong AT, Hickey EC, Ash AS, Berlowitz DR. Identifying hypertension-related comorbidities from administrative data: what's the optimal approach? Am J Med Qual. 2004;19(5):201-6. doi:10.1177/106286060401900504

51. Wilchesky M, Tamblyn RM, Huang A. Validation of diagnostic codes within medical services claims. J Clin Epidemiol. 2004;57(2):131-41. doi:10.1016/S0895-4356(03)00246-4

52. Quan H, Li B, Duncan Saunders L, et al. Assessing validity of ICD-9-CM and ICD-10 administrative data in recording clinical conditions in a unique dually coded database. Health Serv Res. 2008;43(4):1424-41. doi:10.1111/j.1475-6773.2007.00822.x

53. Xu Y, Martin E, D'Souza AG, et al. Enhancing ICD-Code-based case definition for heart failure using electronic medical record data. J Card Fail. 2020;15. doi:10.1016/j.cardfail.2020.04.003

54. Schultz SE, Rothwell DM, Chen Z, Tu K. Identifying cases of congestive heart failure from administrative data: A validation study using primary care patient records. Chron Dis Inj Canada. 2013;33(3):160-6. doi:10.24095/hpcdp.33.3.06

55. Humphries KH, Rankin JM, Carere RG, Buller CE, Kiely FM, Spinelli JJ. Co-morbidity data in outcomes research: are clinical data derived from administrative databases a reliable alternative to chart review? J Clin Epidemiol. 2000;53(4):343-9. doi:10.1016/s0895-4356(99)00188-2

56. Quan H, Parsons GA, Ghali WA. Validity of information on comorbidity derived from ICD-9-CCM administrative data. Med Care. 2002;40(8):675-85. doi:10.1097/00005650-200208000-00007

57. So L, Evans D, Quan H. ICD-10 coding algorithms for defining comorbidities of acute myocardial infarction. BMC Health Serv Res. 2006;6:161-. doi:10.1186/1472-6963-6-161

58. Soo M, Robertson LM, Ali T, et al. Approaches to ascertaining comorbidity information: validation of routine hospital episode data with clinician-based case note review. BMC Res Notes. 2014;7:253-. doi:10.1186/1756-0500-7-253

59. Kaspar M, Fette G, Güder G, et al. Underestimated prevalence of heart failure in hospital inpatients: a comparison of ICD codes and discharge letter information. Clin Res Cardiol 2018;107(9):778-87. doi:10.1007/s00392-018-1245-z

60. Luthi J-C, Troillet N, Eisenring M-C, et al. Administrative data outperformed single-day chart review for comorbidity measure. Internat J Qual Health Care. 2007;19(4):225-31. doi:10.1093/intqhc/mzm017

61. Henderson T, Shepheard J, Sundararajan V. Quality of diagnosis and procedure coding in ICD-10 administrative data. Med Care. 2006;44(11):1011-9. doi:10.1097/01.mlr.0000228018.48783.34

62. Powell H, Lim LLY, Heller RF. Accuracy of administrative data to assess comorbidity in patients with heart disease: an Australian perspective. J Clin Epidemiol. 2001;54(7):687-93. doi:10.1016/S0895-4356(00)00364-4

63. Preen DB, Holman CDAJ, Lawrence DM, Baynham NJ, Semmens JB. Hospital chart review provided more accurate comorbidity information than data from a general practitioner survey or an administrative database. J Clin Epidemiol. 2004;57(12):1295-304. doi:10.1016/j.jclinepi.2004.03.016

64. Sarfati D, Hill S, Purdie G, Dennett E, Blakely T. How well does routine hospitalisation data capture information on comorbidity in New Zealand? N Z Med J. 2010;123(1310):50-61.

65. Chong WF, Ding YY, Heng BH. A comparison of comorbidities obtained from hospital administrative data and medical charts in older patients with pneumonia. BMC Health Serv Res. 2011;11(1):105. doi:10.1186/1472-6963-11-105

66. McKee PA, Castelli WP, McNamara PM, Kannel WB. The Natural History of Congestive Heart Failure: The Framingham Study. N Engl J Med. 1971;285(26):1441-6. doi:10.1056/nejm197112232852601

67. Charlson ME, Pompei P, Ales KL, MacKenzie CR. A new method of classifying prognostic comorbidity in longitudinal studies: development and validation. J Chronic Dis. 1987;40(5):373-83. doi:10.1016/0021-9681(87)90171-8

68. Carlson KJ, Lee DC, Goroll AH, Leahy M, Johnson RA. An analysis of physicians' reasons for prescribing long-term digitalis therapy in outpatients. J Chronic Dis. 1985;38(9):733-9. doi:10.1016/0021-9681(85)90115-8

69. Schellenbaum GD, Rea TD, Heckbert SR, et al. Survival associated with two sets of diagnostic criteria for congestive heart failure. Am J Epidemiol. 2004;160(7):628-35. doi:10.1093/aje/kwh268

70. Hicks KA, Tcheng JE, Bozkurt B, et al. 2014 ACC/AHA Key Data Elements and Definitions for Cardiovascular Endpoint Events in Clinical Trials. Circulation. 2015;132(4):302-61. doi:doi:10.1161/CIR.0000000000000156

71. Curb JD, McTiernan A, Heckbert SR, et al. Outcomes ascertainment and adjudication methods in the women's health initiative. Ann Epidemiol. 2003;13(9, Supplement):S122-S8. doi:10.1016/S1047-2797(03)00048-6

72. Hendel RC, Budoff MJ, Cardella JF, et al. ACC/AHA/ACR/ASE/ASNC/HRS/NASCI/RSNA/SAIP/SCAI/ SCCT/SCMR/SIR 2008 Key Data Elements and Definitions for Cardiac Imaging. Circulation. 2009;119(1):154-86. doi:doi:10.1161/CIRCULATIONAHA.108.191393

73. Schocken DD, Arrieta MI, Leaverton PE, Ross EA. Prevalence and mortality rate of congestive heart failure in the United States. J Am Coll Cardiol. 1992;20(2):301-6. doi:10.1016/0735-1097(92)90094-4

74. Dickstein K, Cohen-Solal A, Filippatos G, et al. ESC Guidelines for the diagnosis and treatment of acute and chronic heart failure 2008 ‡: The Task Force for the Diagnosis and Treatment of Acute and Chronic Heart Failure 2008 of the European Society of Cardiology. Developed in collaboration with the Heart Failure Association of the ESC (HFA) and endorsed by the European Society of Intensive Care Medicine (ESICM). Eur Heart J. 2008;29(19):2388-442. doi:10.1093/eurheartj/ehn309
